# Dendrite morphogenesis in *Caenorhabditis elegans*

**DOI:** 10.1093/genetics/iyae056

**Published:** 2024-05-24

**Authors:** Maxwell G Heiman, Hannes E Bülow

**Affiliations:** Division of Genetics and Genomics, Boston Children's Hospital, Boston, MA 02115, USA; Department of Genetics, Blavatnik Institute, Harvard Medical School, Boston, MA 02115, USA; Department of Genetics, Albert Einstein College of Medicine, Bronx, NY 10461, USA; Dominick P. Purpura Department of Neuroscience, Albert Einstein College of Medicine, Bronx, NY 10461, USA

**Keywords:** dendrite, morphogenesis, cilia, development, glia, adhesion, somatosensory, sensory, WormBook

## Abstract

Since the days of Ramón y Cajal, the vast diversity of neuronal and particularly dendrite morphology has been used to catalog neurons into different classes. Dendrite morphology varies greatly and reflects the different functions performed by different types of neurons. Significant progress has been made in our understanding of how dendrites form and the molecular factors and forces that shape these often elaborately sculpted structures. Here, we review work in the nematode *Caenorhabditis elegans* that has shed light on the developmental mechanisms that mediate dendrite morphogenesis with a focus on studies investigating ciliated sensory neurons and the highly elaborated dendritic trees of somatosensory neurons. These studies, which combine time-lapse imaging, genetics, and biochemistry, reveal an intricate network of factors that function both intrinsically in dendrites and extrinsically from surrounding tissues. Therefore, dendrite morphogenesis is the result of multiple tissue interactions, which ultimately determine the shape of dendritic arbors.

## Introduction

Sensory neurons are how we perceive the world—ultimately, even the most vivid experience is merely a record of the activation patterns of particular sets of sensory neurons. Sensory neurons also exhibit strikingly distinct cellular architecture that is intimately tied to their function. Thus, understanding sensory neuron development opens a window both to the nature of experience and to the fundamental question of how cells attain specific shapes.

The function and organization of sensory neurons are conserved between humans and *Caenorhabditis elegans*. In humans, vision, hearing, taste, and smell are mediated by ciliated sensory neurons that are embedded in discrete organs with specialized glial support cells: vision, by photoreceptors in the retina with Müller glia; hearing, by hair cells in the cochlea with 5 subtypes of support cells; taste, by taste cells in taste buds with so-called Type I support cells; and smell, by olfactory neurons in the olfactory epithelium with sustentacular cells (Roper 2013; Wan *et al*. 2013; Liang 2020; Tworig and Feller 2021). Similarly, in *C. elegans*, diverse environmental cues including tastes, smells, temperature, oxygen, and some aspects of touch are detected by ciliated sensory neurons that are arranged in discrete sense organs together with specialized glia (Ward *et al*. 1975; Goodman and Sengupta 2019; Singhvi and Shaham 2019; Ferkey *et al*. 2021). Indeed, every glial cell in *C. elegans* is found in a sense organ with ciliated sensory neurons, and the glia of each sense organ are molecularly distinct (Fung *et al*. 2020). A central focus of study for ciliated sensory neurons is to understand the roles of these specialized glia in promoting dendrite development and sense organ assembly.

By contrast, in both humans and *C. elegans*, the sense of touch is mediated by nonciliated neurons that have complex branched dendrites (or sensory axons) embedded in the skin. In humans, this includes a diverse array of mechanoreceptive neurons that interact with distinct subdermal structures and preferentially respond to gentle touch, painful touch, or proprioceptive cues (Meltzer *et al*. 2021). Likewise, in *C. elegans*, distinct mechanosensitive dendrites are embedded in different subdomains under the skin (epidermis, also called hypodermis) and respond mainly to gentle or harsh touch or mediate proprioception. As an added complexity, these neurons change throughout postembryonic development and in a specialized life stage called dauer. A central focus of study for these neurons is to understand how they attain their distinctive morphologies and their characteristic attachments to the skin, 2 features that turn out to be intimately coupled.

In *C. elegans*, ciliated sensory neurons and somatosensory neurons have a bipolar morphology, with 2 processes corresponding to a dendrite and axon, unlike most other *C. elegans* neurons that are unipolar, i.e. possess a single process that runs along other processes and forms synapses *en passant*. Other bipolar neurons in *C. elegans* include the touch receptor and motor neurons, which have recently been reviewed (Chisholm *et al*. 2016). In this chapter, we will review the anatomy, development, and genetics of dendrite morphogenesis for ciliated sensory dendrites and the highly branched somatosensory dendrites. An important theme we will emphasize is that dendrite development is fundamentally a product of cell–cell interactions between the neuron and the surrounding cells: glia, skin, muscle, and other neurons. The cellular and molecular steps that give rise to these structures thus provide a model for how developmental cell–cell interactions are used to coordinate the morphogenesis of diverse cellular structures within a tissue.

## Dendrite development of ciliated sensory neurons

*C. elegans* adult hermaphrodites have 62 ciliated sensory neurons that can be divided into 2 classes: 54 neurons have ciliated dendrite endings that are ensheathed by glia (46 in the head, although see note about the FLP neuron below in “Ciliated dendrites not ensheathed by glia”; 8 in the body and tail) while 8 neurons have ciliated dendrite endings that are not glial ensheathed (6 in the head; 2 in the body and tail) (Ward *et al*. 1975; White *et al*. 1986) (Table 1). Almost every subtype of ciliated sensory neuron can be genetically manipulated with remarkable precision using highly cell-type-specific promoters, thus providing a powerful set of tools to visualize individual neurons or alter their function (Table 1).

**Table 1.** Overview of ciliated dendrites.

Neuron	Symmetry	Ciliated ending*^a^*	Glial attachment	Cell-specific promoter*^b^*
***1. Ciliated dendrites ensheathed by glia: 54 neurons^c^***				
**1A. Amphid (AM, 12 neurons + 2 glia)**				
AWA	2	ex, em	AMsh	*odr-10p*
AWB	2	ex, em	AMsh	*str-1p*
AWC	2	ex, em	AMsh	*odr-1p*
AFD	2	em	AMsh	*gcy-8p*
ASE	2	ex	AMsh	*gcy-5p, gcy-7p*
ADF	2	ex	AMsh	*srh-142p*
ASG	2	ex	AMsh	*ops-1p*
ASH	2	ex	AMsh	*osm-10p*
ASI	2	ex	AMsh	*srg-47p*
ASJ	2	ex	AMsh	*gpa-9p*
ASK	2	ex	AMsh	*srbc-64p*
ADL	2	ex	AMsh	*srh-234p*
**1B. Outer labial quadrant (OLQ, 1 neuron + 2 glia)**				
OLQ	4	ex, cu	OLQsh	*ocr-4p*
**1C. Outer labial lateral (OLL, 1 neuron + 2 glia)**				
OLL	2	ex, cu	OLLsh	*ser-2prom3*
**1D. Cephalic (CEP, 1 neuron*^c^* + 2 glia)**				
CEP	4	ex, cu	CEPsh	*dat-1p*
**1E. Inner labial (IL, 2 neurons + 2 glia)**				
IL1	6	ex, cu	ILsh	*flp-3p*
IL2*^d^*	6	ex	ILsh	*klp-6p*
**1F. Anterior deirid (ADE, 1 neuron + 2 glia)**				
ADE	2	ex, cu	ADEsh	*dat-1p*
**1G. Posterior deirid (PDE, 1 neuron + 2 glia)**				
PDE*^c^*	2	ex, cu	PDEsh	*dat-1p*
**1H. Phasmid (PH, 2 neurons*^b^* + 2 glia)**				
PHA	2	ex	PHsh	*nlp-7p*
PHB	2	ex	PHsh	*nlp-1p*
***2. Ciliated neurons that are not ensheathed by glia: 8 neurons^c,e^***				
BAG	2	in	ILsoL/R	*flp-17p*
URX*^e^*	2	in	ILsoL/R*^e^*	*flp-8p*
FLP*^d^*^,*e*^	2	in	ILsoL/R*^e^*	*mec-3p, sto-5p^b^*
AQR	1	in	None	*gcy-33p*
PQR*^c^*	1	in	PHso2L	*gcy-33p*
***3. Somatosensory neurons without cilia***				
PVD	2	n/a	n/a	*F49H12.4p^b^, ser-2prom3^b^, ser-2p3s^f^,* *des-2p^b^*

^*a*^Ciliated endings: ex, exposed to the external environment; em, embedded in lumenal compartment of sheath glia; cu, covered in cuticle sheet; in, internally exposed to pseudocoelomic environment.

^*b*^Cell-specific promoters are mostly restricted to the cell of interest but may also be expressed in a limited number of cells elsewhere. For example, *ser-2prom3* is specific to OLL in the head but is also expressed in PVD in the body; *sto-5p* is also expressed in BDU neurons (Kratz III 2010); *F49H12.4p* is also expressed in AQR and PQR; and *des-2p* is also expressed in FLP and possibly muscle (Zhu *et al*. 2017). In many cases, additional cell-specific promoters are available.

^*c*^Data are shown for adult hermaphrodites. PDE and PQR are born postembryonically and not present in embryos/young L1s. Adult males have an additional ciliated CEM neuron in the cephalic sense organ; ciliated PHD neuron (and possibly cilia remnant in PHC) in the phasmid sense organ; and many ciliated neurons in male-specific tail sense organs.

^*d*^IL2 and FLP have branched dendritic arbors in dauers and adults, respectively.

^*e*^Electron microscopic studies described URX as unciliated and not glial attached and FLP as ciliated and attached to ILsoL/R glia. More recent work has shown that URX is ciliated and attached to ILsoL/R, but the ending of FLP has not been reexamined.

^*f*^ *ser-2p3s* is a shorter variant of *ser-2prom3* that facilitates DNA manipulations while showing similar expression (Ramirez-Suarez *et al*. 2023).

**Fig. 1.**
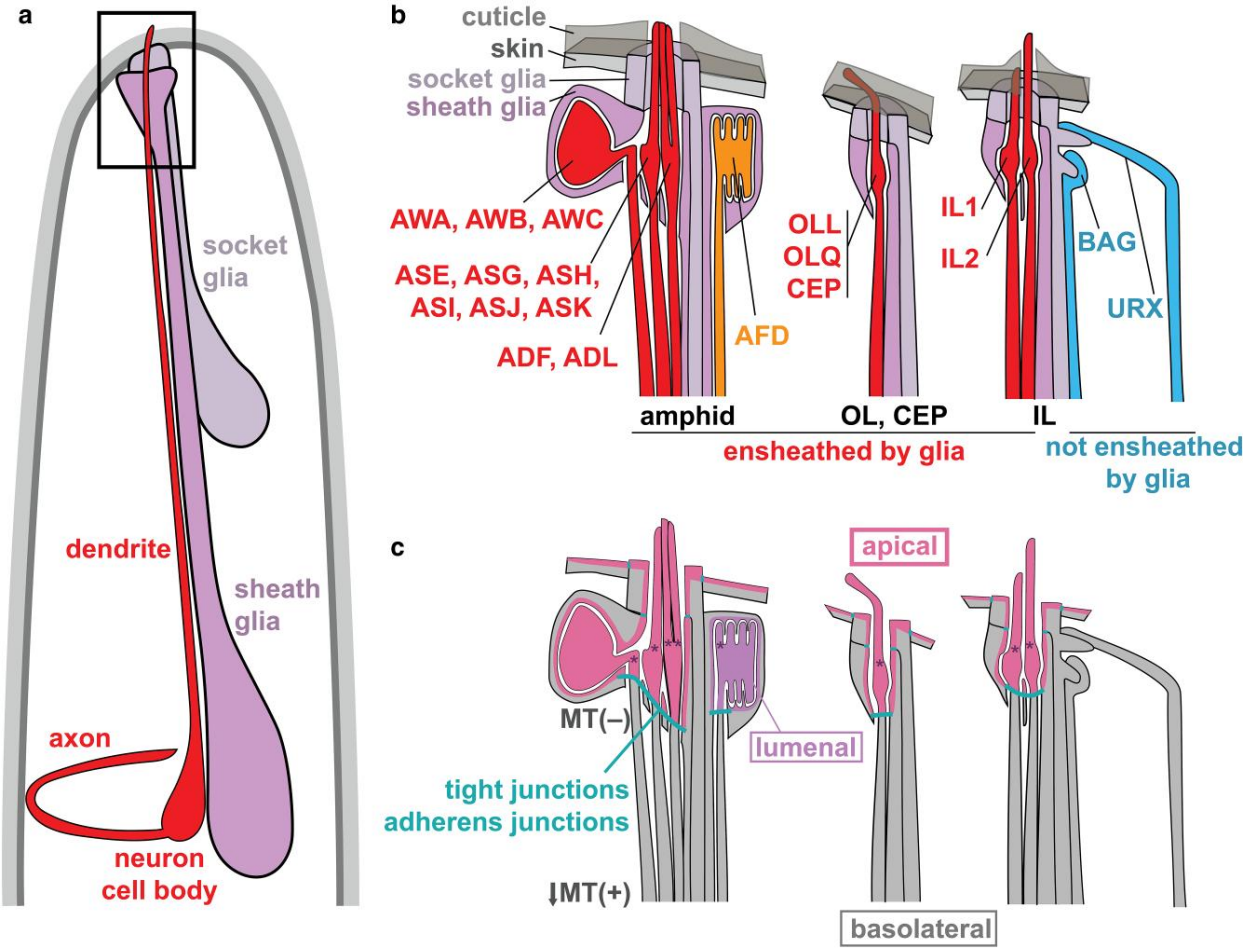
The structure of ciliated dendrites in *C. elegans*. a) Most ciliated dendrites are ensheathed by glial cells in sense organs. Schematic of the head showing a glial-ensheathed sensory dendrite (red). The long unbranched dendrite extends to the nose where it enters an epithelial tube formed by 2 glial cells, called the sheath and socket. b) Schematic of the boxed region in a). Glial-ensheathed ciliated dendrites are organized into the amphid, OL, CEP, and IL sense organs in the head, as well as the ADE, PDE, and phasmid sense organs in the body (not shown). Ciliated endings have direct access to the external environment (singlet cilia, ASE, ASG, ASH, ASI, ASJ, and ASK; doublet cilia ADF, ADL, and IL2), are embedded in cuticle on the external surface of the skin (OLL, OLQ, CEP, and IL1), or are sequestered in intracellular compartments that are open to the external environment (AWA, AWB, and AWC) or not (AFD). Nonensheathed ciliated dendrites include BAG and URX in the head, as well as FLP, AQR, and PQR (not shown), which have direct access to the internal environment of the pseudocoelom. Except AQR, each of their nonensheathed ciliated endings forms a specialized membranous attachment to a glial partner. c) For glial-ensheathed neurons, the glia and dendrites form an epithelial sheet that is continuous with the skin, with tight and adherens junctions delimiting an outward-facing apical surface that is biochemically distinct from the inward-facing basolateral surface. The AFD ending is in a private lumenal compartment that is not open to the internal or external environments. The MT cytoskeleton is also polarized, with minus ends (−) at the cilium BB and plus ends (+) oriented toward the cell body and axon.

In each neuron subtype, the cilium has its own distinctive morphology, molecular receptors, and signal transduction machinery that together determine its sensory repertoire, such as responding to odors, temperatures, or mechanical cues. The diverse functions of ciliated sensory neurons in *C. elegans* were reviewed recently (Goodman and Sengupta 2019; Ferkey *et al*. 2021; Maurya 2022). In mature neurons, the cilium confers not only specific sensory functions but also key aspects of neuron polarity: the ciliary basal body (BB), which is a centriole-derived structure that is positioned at the base of the cilium, serves as a microtubule (MT)-organizing center (MTOC), with the result that dendrite MTs are oriented with their (−) ends positioned distally toward the dendrite ending and their (+) ends positioned toward the cell body (Fig. 1) (Harterink *et al*. 2018).

Prior to cilia formation, each of these neurons undergoes morphogenetic changes that involve a complex series of neuron–glia interactions that establish cell polarity and cilium position and that physically sculpt the dendrite. Here, we will summarize the cellular and molecular steps necessary for dendrite development of ciliated sensory neurons.

## Ciliated dendrites ensheathed by glia

### Glial-ensheathed dendrites are arranged in sense organs

Neurons with glial-ensheathed ciliated endings are arranged in sense organs, also called sensilla. Each sense organ contains from 1 to 12 sensory neurons (1 neuron: OL, CEP, ADE, and PDE; 2 neurons: IL and PH; 12 neurons: AM; Fig. 1) that associate with exactly 2 glial cells, called the sheath and socket glia (Ward *et al*. 1975; Singhvi and Shaham 2019). The bilaterally symmetric amphids are the largest sense organs, accounting for 24 of the 54 glial-ensheathed hermaphrodite neurons, and have received most of the attention to date. Individual amphid neurons mediate responses to soluble (“taste”) and volatile (“smell”) chemical cues, temperature, osmotic conditions, social pheromones, and many other signals (Goodman and Sengupta 2019; Ferkey *et al*. 2021).

### Glial-ensheathed dendrites are topologically part of the skin epithelium

A unifying feature of *C. elegans* sense organs is that the glial cells create a tube-shaped channel that is continuous with the skin and opens to the external environment. By protruding through this channel, the ciliated dendrite endings are positioned on the exterior of the animal where they can sense external cues (Fig. 1). In some cases, the cilium has direct access to the chemical environment through a pore in the cuticle (IL2, ASE, ADF, ASG, ASH, ASI, ASJ, ASK, ADL, PHA, and PHB), while in other cases, the cilium is embedded in the cuticle that covers the animal surface, presumably to sense mechanical force (OLL, OLQ, IL1, CEP, ADE, and PDE) (Ward *et al*. 1975; Doroquez *et al*. 2014) (Fig. 1b). Interesting exceptions to this rule are the 3 pairs of amphid odor-sensing neurons AWA, AWB, and AWC that pass through the amphid glial channel but then veer off into an internal pocket of the sheath glial cell and the amphid temperature-sensing neuron AFD whose ciliated ending is enclosed in a private lumenal compartment of the sheath glial cell that does not have access to the outside (Ward *et al*. 1975; Doroquez *et al*. 2014) (Fig. 1b). Notably, all of the glial-ensheathed cilia—including AWA, AWB, AWC, and AFD—are sealed off from the animal's internal environment by apical junctions, which consist of components homologous to mammalian tight junctions (DLG-1/Discs large) and adherens junctions (HMR-1/Cadherin) (Ward *et al*. 1975; Doroquez *et al*. 2014; Pásti and Labouesse 2014; Low *et al*. 2019) (Fig. 1c).

Topologically, most neurons with glial-ensheathed sensory cilia can be viewed as part of the epithelium of the skin (Heiman 2022). An epithelium is a sheet of cells joined together by tight and adherens junctions to create a diffusion barrier that separates an outward-facing (apical) compartment from an inward-facing (basolateral) compartment. Amphid neurons form junctions with the sheath glial cell, which in turn forms junctions to the socket, which forms junctions to the skin, delimiting a contiguous outward-facing apical surface (Ward *et al*. 1975; Low *et al*. 2019) (Fig. 1c). These intercellular junctions provide a diffusion barrier that separates biochemically distinct membrane compartments within each neuron and glial cell. Apical membrane markers localize to the externally exposed ending of the dendrite and the lumenal surfaces of the glia (Low *et al*. 2019; Lillis *et al*. 2022). Thus, glial-ensheathed ciliated sensory neurons span the apical–basal axis of the skin epithelium, and the neurons and glia themselves exhibit apical–basal polarity in addition to axon–dendrite polarity. Epithelia that contain sensory cells and supporting glia are called sensory epithelia and include the mammalian olfactory epithelium, taste buds, retina, and cochlea (Schlosser 2018; Heiman 2022).

### Development of glial-ensheathed sensory dendrites in the amphid

**Fig. 2.**
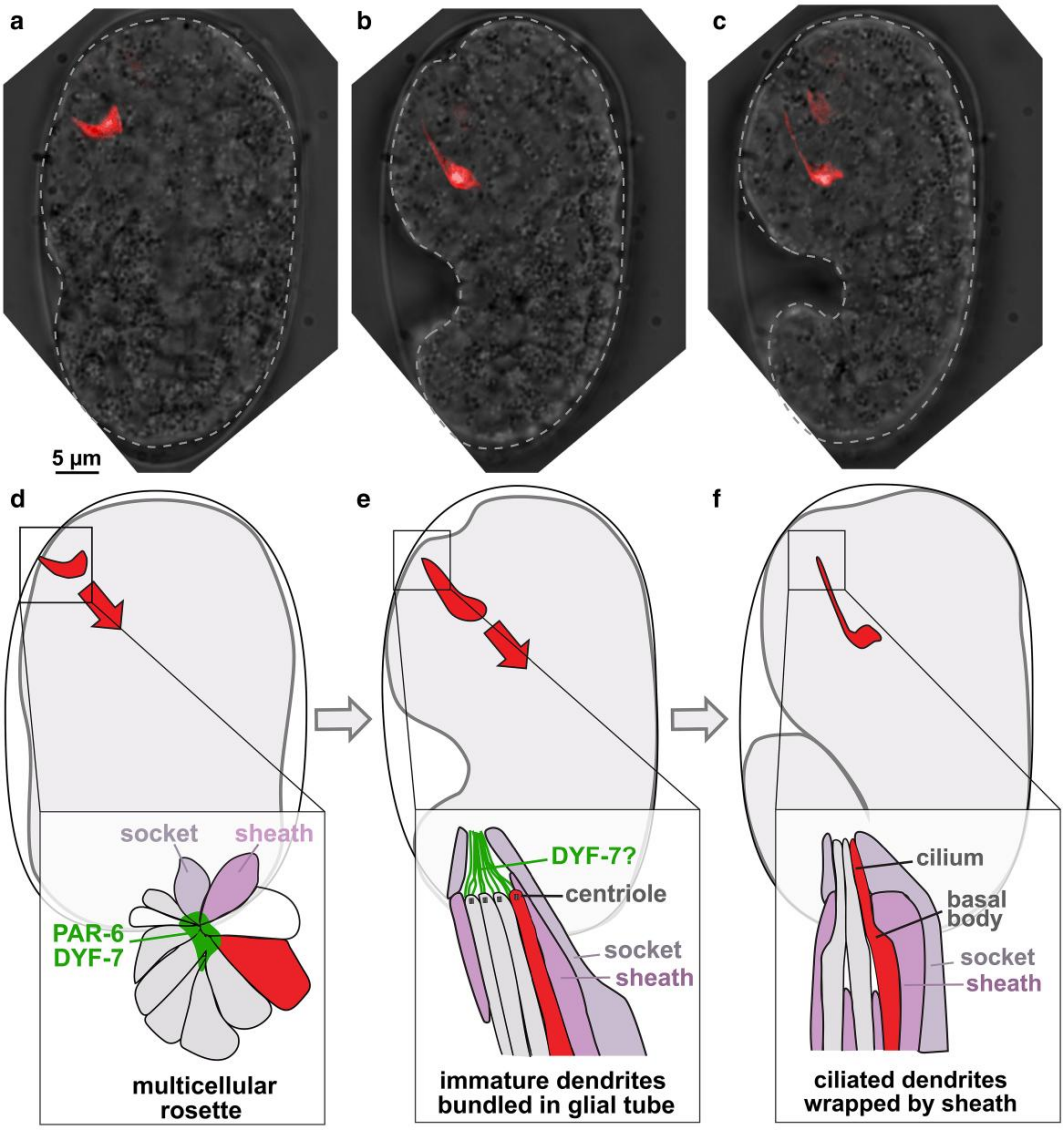
Amphid dendrite formation by retrograde extension. a–c) Time-lapse images of a single amphid neuron in a developing embryo. The neuron is born near the presumptive nose, anchors its dendrite ending there, and then is stretched to its full length. Figure adapted from Heiman and Shaham (2009). Scale bar for a)–c): 5 µm. d) At the rosette stage, the amphid neurons (gray, red) form a multicellular rosette with amphid glia and some nonamphid neurons (white). The apical markers PAR-6 and DYF-7 localize to the vertex of the rosette, suggesting the cells have established polarity at this stage. Insets in d)–f) are not drawn to scale. e) As the dendrites begin to extend, the immature dendrites are bundled as a sheaf within the glial tube. Cilia have not yet formed, and centriole-like structures are visible by electron microscopy at the dendrite endings. A fibrillar matrix that may include DYF-7 extends from the dendrite endings to line the apical surface (lumen) of the glial tube. f) In the mature structure, the sheath glial cell wraps each dendrite individually rather than as a bundle. Centrioles have degenerated, and TZs and cilia have formed. For simplicity, this is shown as corresponding to the comma stage c), when dendrites appear morphologically mature, but it may develop later.

Following proper cell fate specification, the development of glial-ensheathed sensory dendrites can be considered in 3 steps: (1) first, the neurons and glia of a given sense organ recognize and attach to each other and acquire cell polarity; (2) next, the glia undergo morphological changes to form a tube-shaped channel surrounding the dendrite endings; and (3) finally, the dendrites extend by stretch to acquire their mature morphology (Fig. 2). These steps have been studied most intensively in the amphid (Fig. 2a–c).

#### Sorting neurons and glia into sense organs

How sensory neurons and glia recognize the correct partners remains unknown. Unlike in *Drosophila*, the neurons and glia of each sense organ in *C. elegans* are not derived from a single progenitor cell (notable exceptions are the male tail sense organs and PDE, which develop postembryonically, discussed below) (Sulston and Horvitz 1977; Sulston *et al*. 1983). Molecular identity alone does not seem to explain it—there is not a single factor, or combination of factors, that has been identified as distinctive to all members of the same sense organ (Reilly *et al*. 2020). Physical proximity is also not sufficient, because while cells of the same sense organ are typically born near each other, they are also in contact with many other cells that do not become part of the sense organ (Sulston *et al*. 1983). Classical laser ablation experiments, in which entire developmental lineages were deleted, found that some ciliated sensory neurons are able to pair with different socket glia when their normal partners are absent (Sulston *et al*. 1983). This result suggests a graded affinity model of cell recognition, such as differential adhesion among a hierarchy of preferred partners, rather than a lock-and-key model. Differential adhesion has also been proposed to explain the stereotyped positioning of dendrites within the mature amphid bundle, as well as axons in the nerve ring, ventral nerve cord, and male-specific sensory circuits (Kim and Emmons 2017; Yip and Heiman 2018; Hutter 2019; Sengupta *et al*. 2021).

In the amphid, the neurons and glia first assemble into a multicellular rosette (Fan *et al*. 2019) (Fig. 2d). The rosette also includes nonamphid neurons (AUA, AIB, AVB, RIA, and URB) that presumably withdraw from the rosette through unknown mechanisms; interestingly, the more distantly related nematode *Acrobeles complexus* has an “extra” amphid neuron that is proposed to be the homolog of AUA, suggesting that additional rosette neurons have the potential to remain as part of the amphid (Bumbarger *et al*. 2009). At the rosette stage, the amphid neurons and glia are polarized toward a central vertex that exhibits apical identity, shown by localization of the canonical apical determinant PAR-6 and the apical extracellular matrix (aECM) molecule DYF-7 (Fan *et al*. 2019) (Fig. 2d). As the developing skin migrates toward the presumptive nose, it interacts with the central vertex of the rosette and carries it anteriorly toward the future nose tip. This depends on PAR-6 as well as redundant contributions of DYF-7, the cadherin HMR-1, and the adhesion molecule SAX-7/L1CAM (Fan *et al*. 2019). At this stage, cilia have not yet formed but several MT-associated proteins are concentrated at the presumptive dendrite endings, including the centriole-associated protein γ-tubulin (GIP-2), the pericentriolar material-associated proteins SPD-5 and PCMD-1, and, surprisingly, kinetochore proteins (KNL-1, KNL-3, NDC-80, HIM-10, and HCP-4) and the Aurora kinase AIR-2 (Cheerambathur *et al*. 2019; Bai *et al*. 2020; Magescas *et al*. 2021). Depletion of kinetochore proteins delays dendrite extension, while depletion of AIR-2 leads to variable defects in dendrite extension, possibly reflecting roles for MT dynamics at the developing dendrite tip (Cheerambathur *et al*. 2019; Bai *et al*. 2020).

#### Glial wrapping of dendrite endings

Through dramatic morphological rearrangements, the multicellular rosette transforms into a tube-shaped glial channel surrounding the dendrite endings (Fig. 2e). While many details of this rearrangement remain unclear, electron microscopy shows that early in development the dendrites enter the glial tube as a bundle, with junctions directly between the dendrites (Oikonomou *et al*. 2011; Low *et al*. 2019) (Fig. 2e). By contrast, in the mature amphid, each dendrite enters the glial tube individually, with junctions between each dendrite and the sheath but not between neighboring dendrites (Ward *et al*. 1975; Perkins *et al*. 1986; Doroquez *et al*. 2014; Low *et al*. 2019) (Fig. 2f). A possible mechanism could involve processes from the sheath glial cell infiltrating between the bundled dendrites, replacing neuron–neuron contacts with glia–neuron contacts—analogous to radial sorting of axons by nonmyelinating glia in the developing mammalian nervous system (Harty and Monk 2017)—with the sheath glial processes then fusing to each other to create a seamless tube around each dendrite.

Interactions with the sheath glial cell are required for dendrite development, as embryonic ablation of the amphid sheath glial cell prevents dendrite extension (Bacaj *et al*. 2008). Junctions between the amphid dendrites and amphid sheath require the scaffolding protein GRDN-1/CCDC88C for their establishment or maintenance, as *grdn-1/CCDC88C* mutants exhibit mislocalization of the apical junction marker AJM-1 in some amphid neurons, which is accompanied by shortened dendrites that appear to have detached from the sheath glial cell (Nechipurenko *et al*. 2016). GRDN-1/CCDC88C is also required to localize BB and transition zone (TZ) proteins that organize the formation of the sensory cilium, suggesting a relationship between BB/TZ localization and cell junction formation (Nechipurenko *et al*. 2016). During amphid dendrite development, a prominent centriole or BB is visible at each dendrite tip, although cilia have not yet formed (Low *et al*. 2019) (Fig. 2e). Notably, we use the terms “centriole or BB” because it is at this stage that centrioles appear to undergo structural and molecular remodeling to produce ciliary BBs (Nechipurenko and Sengupta 2017). Mutants lacking combinations of BB/TZ components (*ccep-290*, *nphp-1*, *nphp-4*, *mks-1*, *mks-5*, *mks-6*, *mksr-1*, *mksr-2*, and *tctn-1*) exhibit dendrite development defects in the phasmid, a smaller sense organ in the tail (Williams *et al*. 2008, 2011; Schouteden *et al*. 2015; Yee *et al*. 2015). Specifically, they exhibit shortened phasmid dendrites that appear disconnected from the phasmid sheath glial cell, which maintains its normal morphology (Williams *et al*. 2008; Schouteden *et al*. 2015). Together, these observations support the hypothesis that proteins involved in BB/TZ assembly are required to establish or maintain cellular junctions among dendrites or between dendrites and the sheath glial cell, which in turn are required for normal dendrite development.

#### Dendrite extension

Finally, the amphid neuron cell bodies move posteriorly, stretching the dendrites out behind them (Sulston *et al*. 1983; Heiman and Shaham 2009) (Fig. 2). This process of neurite growth by stretch is referred to as retrograde extension, to distinguish it from anterograde extension, in which a migratory growth cone or similar structure drives outgrowth of the neurite tip (see below for further discussion of anterograde dendrite extension for the PQR and PVD neurons, both of which develop postembryonically). Retrograde extension is not unique to *C. elegans*. It has been reported in zebrafish olfactory neuron development, and similar phenomena are observed in mammalian oculomotor axon development, in which neurons lay their axons down behind them as they migrate across the brain midline, and in the development of radial neurites of cerebellar granule cells (Rakic 1971; Puelles-Lobez *et al*. 1975; Puelles 1978; Solecki *et al*. 2004; Breau *et al*. 2017; Monnot *et al*. 2022). More generally, stretch-mediated neurite growth occurs whenever a tissue increases in size. Indeed, as early as 1941, it was observed that, after growing out and attaching to its target, “the nerve is drawn out by the growth and dislocations of its terminal tissues” (Smith 2009; Weiss 2013). In the extreme case of a newborn blue whale, the prodigious rate of organismal growth implies that axons stretch at a rate up to 20 µm/min—at least 10× faster than a growth cone can crawl (Smith 2009)! Retrograde extension in the amphid offers a system for studying the poorly understood mechanisms that underlie neurite anchoring and stretch.

The aECM proteins DEX-1 and DYF-7 are required for amphid dendrite extension by stretch (Heiman and Shaham 2009; Low *et al*. 2019). In *dex-1* or *dyf-7* mutants, all 12 amphid dendrites and the sheath glial cell coordinately detach from the embryonic nose during development, resulting in severely shortened dendrites that extend only 5–10 µm in adults, compared to ∼100 µm in wild-type animals (Heiman and Shaham 2009; Low *et al*. 2019). Despite their length defects, dendrites still develop cilia, form junctions with the sheath glial cell, and exhibit apical–basal polarity; thus, the defects are specific to dendrite extension (Heiman and Shaham 2009; Low *et al*. 2019). In these mutants, the socket glial cell still extends to the nose tip and forms junctions with the skin, but the tube-shaped channel between the sheath and socket glia either fails to form or else ruptures during morphogenesis. Mechanistically, *dex-1* is expressed by many nonneuronal cells, including glia and other epithelia, and encodes a protein with a large extracellular domain containing EGF and nidogen domains and a predicted transmembrane sequence that is dispensable for dendrite extension (Heiman and Shaham 2009). DEX-1 is broadly required for the morphogenesis of diverse epithelial organs, including the excretory system and the pharynx, in which it localizes to apical (lumenal) surfaces (Cohen *et al*. 2019; Flatt *et al*. 2019). *dyf-7* is expressed by sensory neurons and encodes a zona pellucida (ZP) domain protein, part of a protein family that is a nearly ubiquitous component of aECM across tissues and species (Heiman and Shaham 2009; Plaza *et al*. 2010; Cohen and Sundaram 2020). DYF-7 localizes to “caps” at the dendrite endings and, when misexpressed in other tissues, localizes to apical surfaces (Low *et al*. 2019). Like other ZP domain proteins, DYF-7 is synthesized with a transmembrane sequence, undergoes proteolytic cleavage to be shed from the membrane, and can form dimers that then multimerize (Heiman and Shaham 2009). When expressed in vitro, fluorescently tagged DYF-7 forms micron-scale filaments that are readily visualized (Low *et al*. 2019). Electron microscopy of the developing amphid at the stage of dendrite extension reveals extracellular filaments extending from the dendrite tips to line the lumen of the glial tube (Oikonomou *et al*. 2011; Low *et al*. 2019) (Fig. 2e). These filaments are absent in *dyf-7* mutants, in which the glial tube ruptures or fails to form (Low *et al*. 2019). Notably, the integrity of narrow epithelial tubes in the *Drosophila* trachea and the *C. elegans* excretory system require lumenal aECM proteins (Sundaram and Cohen 2017; Li Zheng *et al*. 2020). Similarly, DYF-7 is a filament-forming aECM protein that maintains the integrity of a narrow glial tube during morphogenesis. In *dex-1* and *dyf-7* mutants, loss of integrity of this glial tube corresponds to detachment of the dendrites from the developing nose and failure of the dendrites to undergo stretch-mediated growth.

### Development of glial-ensheathed dendrites in other sense organs

Other sense organs, including OL, IL, CEP, and the phasmid, share the same anatomical organization as the amphid, namely the presence of a glial tube surrounding the dendrite endings with tight and adherens junctions that separate an outward-facing apical surface from an inward-facing basolateral surface (Ward *et al*. 1975; Perkins *et al*. 1986). Thus, each of these organs can be viewed as a miniature sensory epithelium continuous with the skin. Their development has not been investigated in depth, but features that have been examined are similar to those of the amphid. For example, the CEP sheath glial cell is required for CEP dendrite extension, and DYF-7 is required by each of these sense organs for dendrite extension (Yoshimura *et al*. 2008; Low *et al*. 2019).

The PDE sense organ has been examined in detail because it arises postembryonically, facilitating direct visualization of its development. The PDE neuron, sheath, and socket glial cells are derived from a single progenitor, together with another neuron (PVD, described below) that will not be part of the mature sense organ (Sulston and Horvitz 1977; Way and Chalfie 1989). Similar to amphid development, these neurons and glia initially form a multicellular rosette, from which the extra neuron eventually withdraws (Lee *et al*. 2021). The apical polarity of the PDE dendrite is inherited from its epithelial precursors, reminiscent of the way in which apical polarity in the amphid neurons is inherited from the rosette stage (Fan *et al*. 2019; Lee *et al*. 2021). This inherited apical polarity is important for establishing features of the mature PDE dendrite, including the positioning of the sensory cilium and the polarized orientation of dendrite MTs (Lee *et al*. 2021).

In summary, a shared feature of glial-ensheathed ciliated dendrites is that they develop “tip first,” starting with the organization of the dendrite ending. This reflects the importance of apical polarization in controlling the positioning of MT-associated proteins, the formation of dendrite–glia junctions, the localization of aECM proteins that maintain integrity of the developing glial tube, and ultimately the establishment of mature features including the cilium. In this way, the development of glial-ensheathed dendrites may more closely resemble the formation of mammalian sense organs from epithelial placodes than it does the outgrowth of the highly branched dendrites described below in “Somatosensory dendrites.”

## Ciliated dendrites not ensheathed by glia

### Nonensheathed dendrite endings have direct access to the pseudocoelom

In addition to the 54 glial-ensheathed neurons discussed above, there are 8 neurons with ciliated dendrites that are not ensheathed by glia: FLP, URX, and BAG are present as 6 bilaterally paired neurons in the head, and AQR and PQR are single neurons in the body and tail, respectively (White *et al*. 1986). Most of these neurons are gas sensors, responding primarily to changes in the concentration of oxygen (URX, AQR, and PQR) or carbon dioxide (BAG) (Carrillo and Hallem 2015). Importantly, because these dendrites do not form tight junctions with glia, their ciliated endings are directly exposed to the pseudocoelom and are ideally positioned to detect changes in the animal's internal oxygen and carbon dioxide levels, analogous to the mammalian carotid body (Fig. 1b). The sensory function of the FLP cilium, if any, is unknown; its dendrite undergoes postembryonic branching to mediate touch responses that have been the focus of study (see “Somatosensory dendrites”).

### Some nonensheathed dendrites form specialized attachments to specific glial partners

**Fig. 3.**
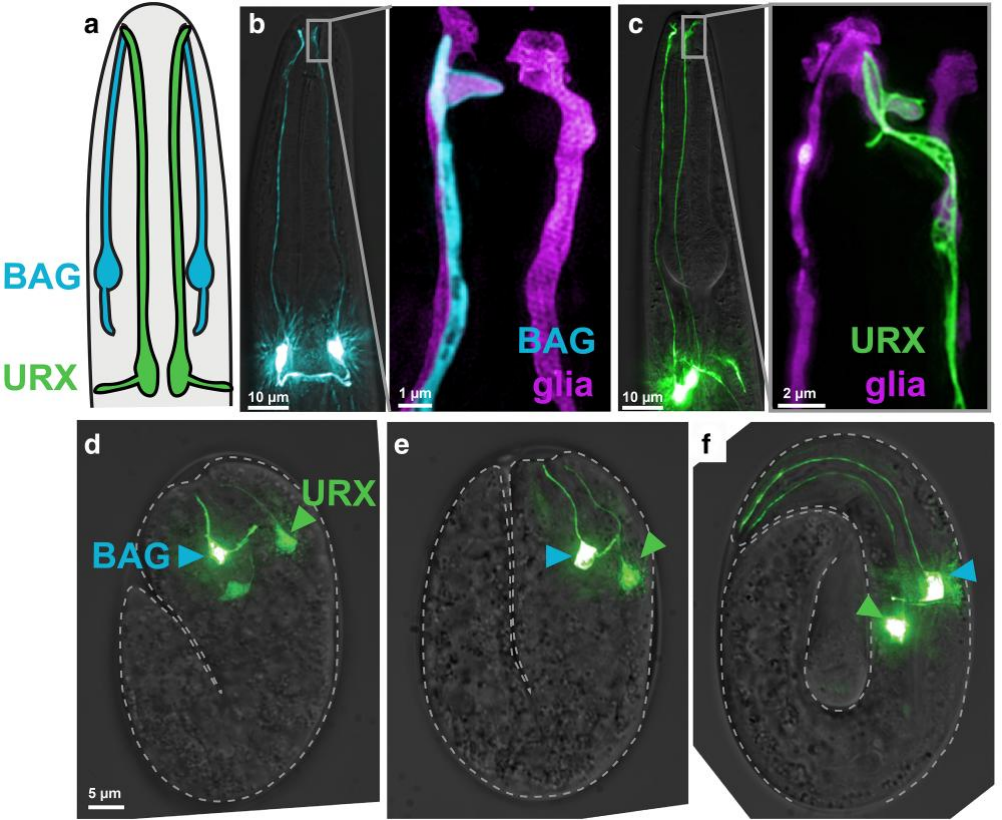
BAG and URX dendrites form specialized attachments to a glial partner and grow by retrograde extension. a) Schematic of the head showing bilateral BAG and URX neurons. The BAG dendrites are positioned laterally, while the URX dendrites are positioned dorsally and “jump” to the lateral position at the nose tip. b, c) Fluorescence images of BAG and URX. Insets, super-resolution structured illumination microscopy images of the b) BAG and c) URX endings with the ILso glial partner. BAG associates with the lateral ILso glial cell, while URX associates with the dorsal nerve bundle and then “jumps” at the nose to contact the lateral ILso glial cell. Low-magnification and insets are different animals; boxed area is meant to illustrate approximately the region shown in the inset. Figure adapted from Cebul *et al*. (2020). d–f) Images of BAG and URX neurons in 3 embryos arranged as a pseudo-time course. The neuron anchors its dendrite ending near the nose and then is stretched to its full-length during embryo elongation. Figure adapted from Cebul *et al*. (2020).

Although they are not positioned within a glial tube, several of these dendrites nevertheless form specialized attachments to specific glia. The BAG dendrite extends along the lateral sensory nerve bundle and, at the nose tip, its cilium forms a membranous “bag” that wraps a protrusion of the lateral IL socket (ILso) glial cell (Ward *et al*. 1975; Doroquez *et al*. 2014; Cebul *et al*. 2020) (Fig. 3a and b). The URX dendrite extends along the dorsal sensory nerve bundle and then projects laterally at the nose tip to form a sheet-like membranous attachment to the same ILso glial cell that is wrapped by BAG (Cebul *et al*. 2020) (Fig. 3a and c) (note: The URX-ILso attachment was identified using super-resolution fluorescence imaging with URX and ILso-specific markers (Cebul *et al*. 2020), but it had not been described in earlier EM reconstructions (Ward *et al*. 1975; Doroquez *et al*. 2014). Those EM studies attributed a ciliated dendrite ending interacting with ILso to be that of FLP, raising the question of whether the URX dendrite ending may have been misassigned. URX was only later shown to be ciliated (Kazatskaya *et al*. 2020). It remains ambiguous if FLP is ciliated; notably, single-cell transcript profiling revealed expression of the cilia gene *osm-6* in all known ciliated neurons except FLP (Taylor *et al*. 2021). The significance of the shared attachment of BAG and URX to the same glial partner remains unclear; however, this arrangement is conserved in the nematode *Pristionchus pacificus*, which diverged from *C. elegans* ∼100 million years ago, suggesting it may have a functional consequence (Hong *et al*. 2019). Finally, in the tail, PQR was described by classical EM studies to embed its cilium in one of the phasmid socket glial cells (PHso2L) (Hall and Russell 1991). Because they are not epithelial, the attachments of the BAG, URX, and PQR ciliated dendrite endings with specific glia may more closely resemble neuron–glia attachments in the mammalian brain, for example between dendritic spines and astrocytic glia.

### BAG and URX dendrites anchor at the nose via glial partners and then extend by stretch

By the 1.5-fold stage of embryogenesis, BAG and URX have extended ∼5- to 10-µm-long dendrites toward the nose tip (Cebul *et al*. 2020). Then, as the embryo elongates, their dendrites stretch 10-fold, reaching ∼50–60 µm in length in pretzel-stage embryos (Cebul *et al*. 2020) (Fig. 3d–f). The mechanism of BAG and URX dendrite stretch is different than that of the amphid. For example, BAG and URX do not require either *dyf-7* or *dex-1*, consistent with their dendrite endings not being anchored within a glial tube (Cebul *et al*. 2020). Genetic screens identified *grdn-1/CCDC88C*, *sax-7/L1CAM*, and *magi-1/MAGUK* as required for BAG and URX dendrites to grow by stretch during embryo elongation (Cebul *et al*. 2020).

GRDN-1/CCDC88C is a ubiquitously expressed cytoskeletal scaffolding protein that controls centriole positioning in ciliated neurons, where it affects the development of some amphid dendrites; however, it acts in glial cells to nonautonomously promote BAG and URX dendrite extension (Nechipurenko *et al*. 2016; Cebul *et al*. 2020). While *grdn-1/CCDC88C* null animals are inviable, hypomorphic alleles of *grdn-1/CCDC88C* completely prevent normal URX dendrite extension and strongly disrupt BAG dendrite extension. In *grdn-1/CCDC88C* mutants, BAG and URX dendrites initially appear normal in 1.5-fold embryos, but, as the embryo elongates, the dendrite endings detach from the developing nose and fail to stretch to their full lengths (Cebul *et al*. 2020). The resulting mature dendrites are ∼50–75% of their normal lengths, with URX more severely affected than BAG (Cebul *et al*. 2020). Glial-specific expression of GRDN-1/CCDC88C is sufficient to rescue URX defects, suggesting that URX dendrite endings may anchor to a glial partner at the nose during embryo elongation.

SAX-7/L1CAM is a single-pass transmembrane adhesion molecule that plays numerous roles in nervous system development and maintenance, including positioning of neuronal cell bodies and axons, lateral bundling of neurites (fasciculation), synapse regulation, and dendrite branching as discussed below (Zallen *et al*. 1999; Bénard *et al*. 2012; Dong *et al*. 2013; Salzberg *et al*. 2013; Opperman *et al*. 2015; Yip and Heiman 2016; Chen *et al*. 2019; Ramirez-Suarez *et al*. 2019). *sax-7/L1CAM* mutants strongly disrupt BAG and URX dendrite extension, with more severe effects on BAG than URX, in contrast to *grdn-1/CCDC88C* mutants (Cebul *et al*. 2020). As in *grdn-1/CCDC88C* mutants, *sax-7/L1CAM* mutant dendrites detach from the embryonic nose during embryo elongation, leading to shortened dendrites that fail to reach their normal positions at the nose. Restoring SAX-7/L1CAM expression in neurons or glia results in partial rescue of dendrite defects, while restoring SAX-7/L1CAM in both neurons and glia together results in nearly complete rescue, consistent with the hypothesis that neuron–glia adhesion via SAX-7/L1CAM homophilic interactions is required to anchor BAG and URX dendrite endings at the developing nose (Cebul *et al*. 2020). A role for SAX-7/L1CAM in anchoring developing dendrites to glia is reminiscent of its role in promoting attachment of somatosensory dendrites to the skin, as discussed in “Higher-order branches grow along an epidermal scaffold.” However, unlike dendrite–skin interactions, dendrite–glia anchoring requires protein interaction motifs in the SAX-7/L1CAM cytoplasmic tail that bind MAGI-1/MAGUK and other factors (Cebul *et al*. 2024).

MAGI-1/MAGUK is a multi-PDZ domain scaffolding protein that was identified in candidate-based genetic screens for mutants with BAG and URX dendrite defects (Cebul *et al*. 2024). Loss of MAGI-1/MAGUK affects both BAG and URX dendrite extension, with more severe effects on URX, while it has minimal or no effect on the lengths of amphid or other ciliated dendrites (Cebul *et al*. 2024). Expression of MAGI-1/MAGUK in glia, but not neurons, is sufficient to rescue BAG and URX dendrite extension defects (Cebul *et al*. 2024). MAGI-1/MAGUK physically binds the SAX-7/L1CAM cytoplasmic tail through a PDZ-binding motif at the SAX-7/L1CAM C-terminus and can simultaneously bind the β-catenin HMP-2, potentially bridging together an adhesion structure that includes both SAX-7/L1CAM and the HMR-1/HMP-1/HMP-2 cadherin/catenin complex (Cebul *et al*. 2024). Consistent with this hypothesis, glial-specific depletion of HMR-1/Cadherin also results in shortened BAG and URX dendrites (Cebul *et al*. 2024).

In summary, SAX-7/L1CAM physically and genetically interacts with MAGI-1/MAGUK and the HMR-1/HMP-1/HMP-2 cadherin/catenin complex in glia to promote BAG and URX dendrite extension by anchoring the nascent dendrite endings at the nose tip. GRDN-1/CCDC88C similarly acts in glia to promote BAG and URX dendrite tip anchoring through an undetermined mechanism. GRDN-1/CCDC88C, SAX-7/L1CAM, MAGI-1/MAGUK, and the HMR-1/HMP-1/HMP-2 cadherin–catenin complex are all conserved components of epithelial adherens junctions, raising the possibility that an adherens junction-like structure in glia anchors the BAG and URX dendrite endings at the embryonic nose during stretch-based growth (Cebul *et al*. 2024).

### AQR and PQR ciliated dendrites form by anterograde outgrowth, not by stretch

The AQR and PQR neurons are born postembryonically from cells of the QR and QL lineages, which undergo long-range migrations to the head and tail, respectively (Rella *et al*. 2016; Chai *et al*. 2018). In L1 larvae, AQR and PQR extend their dendrites by outgrowth ∼9 h after hatching (Chai *et al*. 2018). PQR dendrite outgrowth is guided by Wnt signaling (Kirszenblat *et al*. 2011). The Wnt LIN-44 is expressed in tail skin cells (hyp8, 9, 10, and 11), while the Wnt receptor LIN-17/Frizzled is expressed by PQR (Kirszenblat *et al*. 2011). Loss of LIN-44/Wnt or LIN-17/Frizzled results in shortened, absent, or misdirected PQR dendrites (Kirszenblat *et al*. 2011). The guidance mechanisms controlling the outgrowth of the AQR dendrite are not known. During AQR and PQR dendrite outgrowth, the cytoskeletal organizer GRDN-1/CCDC88C localizes to the growing dendrite tip (Nechipurenko *et al*. 2021). In *grdn-1/CCDC88C* mutants, AQR and PQR dendrites are often shortened, misdirected, or aberrantly branched (Nechipurenko *et al*. 2021). The cilium is also mispositioned, sometimes emerging from the cell body, or absent (Nechipurenko *et al*. 2021). These phenotypes are similar to those seen in amphid neurons in *grdn-1/CCDC88C* mutants (Nechipurenko *et al*. 2016). As in the amphid, GRDN-1/CCDC88C expression in neurons is sufficient to rescue dendrite morphogenesis and cilia positioning defects, suggesting GRDN-1/CCDC88C acts cell autonomously in this context (Nechipurenko *et al*. 2016, 2021). During PQR dendrite outgrowth, the centriolar protein SAS-6 also localizes to the growing dendrite tip (Li *et al*. 2017). However, centriole positioning in the dendrite tip is not required for dendrite outgrowth (Li *et al*. 2017). Rather, centriole translocation slightly lags dendrite outgrowth, and mutants in the dynein-1 components DHC-1, DLC-1, and LIS-1 disrupt centriole translocation but not dendrite outgrowth (Li *et al*. 2017). After the dendrite reaches its full length, SAS-6 at the centriole is replaced with mature TZ markers, such as MKS-5, as the cilium develops (Li *et al*. 2017). In summary, GRDN-1/CCDC88C at the dendrite tip may confer aspects of dendrite polarity that are required both for dendrite outgrowth and for positioning of the centriole and future cilium.

### Maintenance, aging, and degeneration of ciliated dendrites

While this review focuses on how dendrite morphology is initially established, another interesting set of mechanisms govern how it is maintained in the face of organismal growth, movement, aging, and stress. In *C. elegans*, body movement imposes sufficient mechanical force to cause neurite mispositioning or breakage in the absence of ECM, cell adhesion, and cytoskeleton proteins that counteract these forces (Aurelio *et al*. 2002; Bülow *et al*. 2004; Sasakura *et al*. 2005; Wang *et al*. 2005; Bénard *et al*. 2006). Specifically, the gigantic ECM protein DIG-1 and the cell adhesion molecules SAX-7/L1CAM, PTP-3/LAR, and the Fat-like cadherin CDH-4 are required to maintain dendrite fasciculation in the amphid and other ciliated dendrite bundles (Burket *et al*. 2006; Yip and Heiman 2018; Chong *et al*. 2021). Moreover, dendrite breakage is observed in many ciliated dendrites in the head upon loss of the cytoskeletal protein UNC-70/β-Spectrin (Hammarlund *et al*. 2007) and specifically in the BAG dendrite upon loss of DIG-1 (Chong *et al*. 2021). Unlike *C. elegans* axons, which can usually regenerate after breakage, most ciliated dendrites show limited capacity for regrowth, possibly reflecting the lack of a role for anterograde extension in their development (Chung *et al*. 2006, 2016).

The sensory endings of ciliated dendrites can undergo dynamic changes in their morphology, including extensive remodeling during dauer. Dauer-specific remodeling ranges from drastic expansion of the AWC cilia, concomitant with fusion of the left and right amphid sheath glia, to stereotyped branching of the IL2 dendrites (Albert and Riddle 1983; Procko *et al*. 2011; Schroeder *et al*. 2013; Britz *et al*. 2021). Sensory endings of ciliated dendrites also exhibit age-dependent remodeling, including increased complexity in URX, reduced complexity in AFD, and altered compartmentalization requirements in CEP (Cohn *et al*. 2020; Huang *et al*. 2020; Acker *et al*. 2021). Loss of the MAP kinase MAPK-15 leads to extreme overgrowth of the URX dendrite during late larval and adult stages, suggesting the presence of mechanisms that normally restrict dendrite growth in adults (McLachlan *et al*. 2018). Perhaps most remarkably, aging can suppress amphid cilia defects caused by the loss of cilia trafficking components, further emphasizing that there may be mechanisms for dendrite and cilia morphogenesis that are specific to distinct life stages (Cornils *et al*. 2016).

Finally, exposure to toxins and pathogens also affects the maintenance of ciliated dendrite morphology. In particular, dendrite degeneration (including blebbing and breakage) of the dopaminergic CEP neurons has been widely used as a model for Parkinson's disease genes and the effects of pharmacological and environmental neurotoxins such as 6-hydroxydopamine, heavy metals, and microbial-derived agents (see, e.g. Caldwell *et al*. 2009; González-Hunt *et al*. 2014; Caldwell *et al*. 2020; Clark *et al*. 2024). Changes in dendrite morphology are also observed in amphid AWC neurons upon exposure to bacterial pathogens such as *Pseudomonas aeruginosa*, a phenomenon that has also been used as a model of neurodegeneration (Wu *et al*. 2015; Kaur and Aballay 2020; Kaur *et al*. 2022).

Together, these studies underline that dendrite morphology is not static but rather can be remodeled through mechanical force, life stage, and environmental exposures. This theme is further elaborated below in “Plasticity of branched dendritic arbors.”

## Somatosensory dendrites

### PVD, FLP, and IL2 neurons form elaborately branched dendrites

#### Overview and morphogenesis of PVD

The most complex neuronal morphologies in *C. elegans* are found in somatosensory neurons such as the PVD and FLP neuron pairs and IL2 neurons. These neurons are characterized by morphologically distinct and highly elaborate dendritic arbors. The PVD neurons are a bilaterally symmetric pair of cells located in the lateral epidermis, posterior to the vulva, and develop from postembryonic divisions of the V5 epidermal blast cell at the beginning of the L2 larval stage (Sulston and Horvitz 1977). Their extensively branched dendrites were first noticed in immunohistochemical stains against the acetylcholine receptor subunit DEG-3 (Halevi *et al*. 2002) and transgenic animals expressing GFP from a PVD-specific promoter (Tsalik *et al*. 2003).

**Fig. 4.**
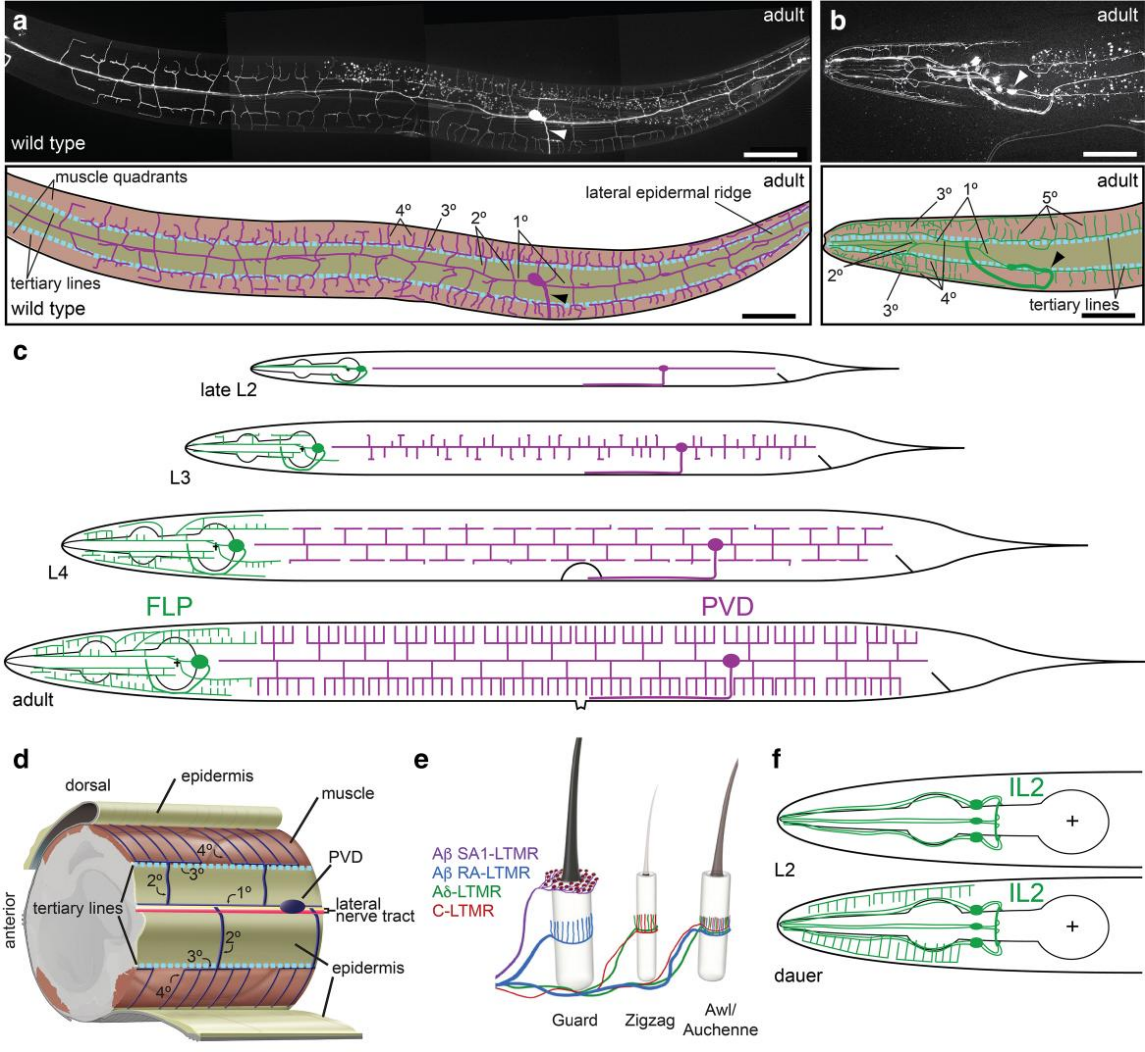
The structure of highly branched somatosensory neurons in *C. elegans*. a, b) Composite epifluorescent micrographs of animals in which PVD a) or FLP b) are fluorescently labeled by GFP (PVD: *wdIs52*, Smith *et al*. 2010; FLP: *dzIs117*, Rahman *et al*. 2022). Note that the *dzIs117* strain also labels a pharyngeal neuron. Schematics with tracings are shown underneath. A light blue dashed line denotes the tertiary lines, i.e. the line where the muscle quadrants and the lateral epidermis abut. Arrowheads indicate axons. Scale bars: 50 µm. Panel a) reuses a fluorescent micrograph from Salzberg *et al*. (2013). c) Schematics showing the development of FLP (green) and PVD (red) neurons during larval stages. Note that while FLP is born embryonically and PVD postembryonically, morphogenesis of the elaborate dendritic arbors of FLP occurs during larval stages, largely concomitant with the development of PVD dendrites. d) Schematic showing the tissue environs of PVD neurons with primary (1°), secondary (2°), tertiary (3°), and quaternary (4°) dendrites indicated. The lateral nerve tract contains besides the 1° PVD dendrite the processes of ALA and CAN neurons (red and yellow). A dashed light blue line indicates the tertiary line, which demarcates where the lateral epidermis/skin (tan) and the muscle quadrants (red-brown) adjoin. This is also where the cell adhesion molecule SAX-7/L1CAM localizes. Figure adapted from Salzberg *et al*. (2013). e) Schematics of the hairy mouse skin showing the innervation and structures of different low threshold mechanosensory receptor neurons (AβSA1-LTMRs, AβRA-LTMRs, Aδ-LTMRs, and C-LTMRs), which innervate different types of sensory hairs. Note the lanceolate structures of AβRA-LTMRs, Aδ-LTMRs, and C-LTMRs neurons that surround the follicles of different hair types and display characteristic right angles. Figure adapted from Li *et al*. (2011). f) Schematics showing the IL2 neurons during either the L2 larval stage (left panel) or the dauer alternative stage when highly elaborate dendritic trees are formed.

Morphogenesis of the PVD dendritic arbor is a highly choreographed process that is temporally controlled by the conserved *lin-4/lin-14* and the *lin-28/let-7/lin-41* regulatory circuits (Suzuki *et al*. 2022). During the L2 larval stage, each PVD neuron sends a commissural axon into the right ventral nerve cord where they turn anteriorly and extend together to a location posterior to the vulva (White *et al*. 1986). At the same time, a primary dendrite extends anteriorly and posteriorly along the lateral nerve tract (Fig. 4a–c) (Oren-Suissa *et al*. 2010; Smith *et al*. 2010; Albeg *et al*. 2011). Subsequently, orthogonal secondary branches extend both dorsally and ventrally toward the edge of the lateral epidermis, where they bifurcate to form orthogonal tertiary dendrites at the boundary of the lateral epidermis and the respective dorsolateral and ventrolateral muscle quadrants, respectively (Fig. 4a–c). These, in turn, form orthogonal quaternary dendrites that grow embedded between a thin epidermal sheet and a basement membrane covering the muscle (Albeg *et al*. 2011). The result of these consecutive orthogonal branching events is highly stereotyped dendritic arbors, which, owing to their resemblance to candelabras, have also been called menorahs (Fig. 4a–c) (Oren-Suissa et al. 2010). While such orthogonal branching may appear unusual, there is precedent for similar morphologies in vertebrates. For example, the peripheral axonal endings of low-threshold mechanosensory neurons in the hairy skin of mammals are also characterized by orthogonal branching (Fig. 4e) (García-Añoveros *et al*. 2001; Li *et al*. 2011).

PVD neurons are polymodal somatosensory neurons because of their involvement in the sensation of harsh touch, proprioception, extreme temperatures, and sound (Way and Chalfie 1989; Chatzigeorgiou *et al*. 2010; Albeg *et al*. 2011; Mohammadi *et al*. 2013; Tao *et al*. 2019; Iliff *et al*. 2021). The dendritic arbors of the PVD bilateral neurons cover the entire body surface of the animal from tail to neck, i.e. excluding the head, which is covered by the dendrites of the pair of FLP or IL2 neurons in a nonoverlapping fashion (Smith *et al*. 2010) (see below). For additional reviews of PVD development, see also Sundararajan *et al*. (2019b) and Inberg *et al*. (2019).

#### Comparison of PVD with FLP and IL2

The FLP neurons are structurally similar to PVD neurons, with primary, secondary, tertiary, and quaternary branches, although the dendritic arbors appear more variable and display some differences compared to PVD dendrites (Fig. 4a–c). First, in contrast to PVD, the FLP neurons are born embryonically although they start to elaborate their dendritic arbors only later within the same time frame as the PVD dendritic arbors between the L2 and L4 larval stages (Albeg *et al*. 2011; Androwski *et al*. 2020). The trajectories of higher-order dendritic branches of FLP neurons differ and more resemble the trajectories of IL2 neurons (discussed below). Specifically, the FLP primary dendrites are positioned along the lateral dendritic fascicle of sensory neurons and, near the metacorpus of the pharynx, secondary processes branch off to grow along the subdorsal and subventral sensory neuron fascicles (Androwski *et al*. 2020). The secondary dendrites send tertiary processes to the dorsal and ventral midlines, where they branch to send perpendicular branches laterally, sandwiched between skin and muscle. At the same time, the dendritic branches along the sublateral lines also branch toward the dorsal and ventral lines to form candle-like arbors (Fig. 4a–c) (Androwski *et al*. 2020). Second, in contrast to PVD neurons, the primary dendrites of FLP neurons may contain ciliated anterior endings, which are not exposed to the outside (Ward *et al*. 1975), although see discussion in “Ciliated dendrites not ensheathed by glia.” The FLP neurons serve related as well as distinct functions compared to PVD neurons. Both FLP and PVD neurons are nociceptive, but FLP neurons sense heat and humidity whereas PVD neurons sense hot and cold temperature and play a role in proprioception and the detection of sound (Chatzigeorgiou *et al*. 2010; Albeg *et al*. 2011; Mohammadi *et al*. 2013; Wang *et al*. 2014; Tao *et al*. 2019; Iliff *et al*. 2021).

The IL2 (inner labial neurons) are a group of 6 bipolar neurons with 6-fold radial symmetry, which are part of the inner labial sensillae (Ward *et al*. 1975; White *et al*. 1986). They are divided into a dorsal, a lateral, and a ventral pair and send unbranched dendrites toward the nose of the animals, where their ciliated endings are exposed to the environment as discussed in “Ciliated dendrites ensheathed by glia” (Fig. 1) (Ward *et al*. 1975). In addition, the IL2s all send a short axonal process into the nerve ring (Fig. 4f). The IL2 neurons are required for nictation behavior in dauer animals (Lee *et al*. 2011) and function as presumptive taste receptors (Perkins *et al*. 1986). Unbranched in favorable environmental conditions, the dendrites of IL2 neurons start forming elaborate dendritic arborizations similar to FLP neurons upon entry into the alternative dauer stage (Schroeder *et al*. 2013; Androwski *et al*. 2020). Specifically, the dorsal and ventral pairs of IL2s grow secondary dendrites orthogonally toward the dorsal and ventral midlines respectively, where they turn perpendicularly in both an anterior and posterior direction to form tertiary dendrites. These tertiary dendrites grow orthogonal quaternary dendrites from the midlines in a lateral direction, sandwiched between the muscle quadrants and the overlying skin in much the same way as the FLP neurons, i.e. in the opposite direction compared to the PVD neurons (Schroeder *et al*. 2013; Androwski *et al*. 2020). The dendritic arborizations of IL2 neurons are reversible and disappear over time upon exit of the animals from the dauer stage (Schroeder *et al*. 2013). In summary, PVD and FLP dendrites develop during larval stages to cover the entire body surface of adult animals, with FLP covering the head and neck and the PVD dendrites covering the remainder of the body. If animals enter the alternative larval dauer stage in unfavorable environmental conditions, IL2 rather than FLP neurons form elaborate dendritic trees to cover the head and neck region (Androwski *et al*. 2020) whereas PVD neurons arrest dendrite morphogenesis just prior to the elaboration of quaternary dendrites (Richardson *et al*. 2019).

#### Tiling and the control of field size of PVD and FLP neurons

**Fig. 5.**
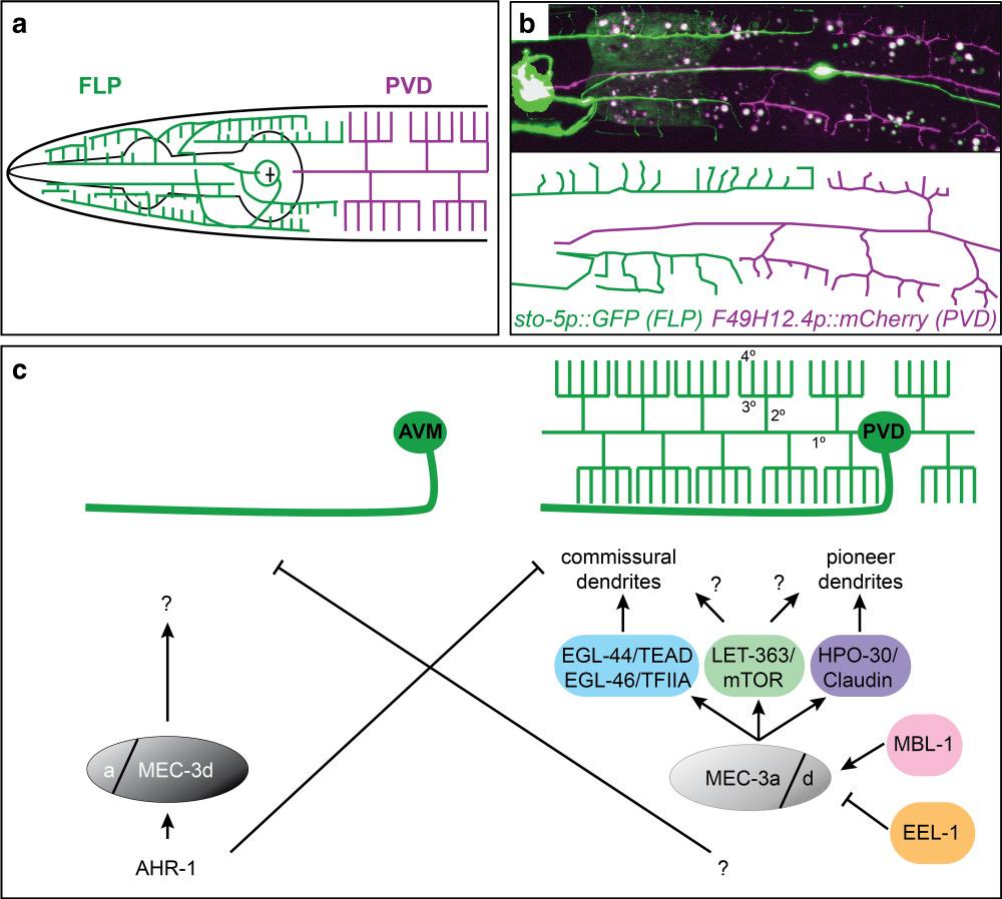
Tiling and morphogenesis of FLP and PVD somatosensory dendrites. a) Schematic of FLP and PVD neurons tiling in the head region of the animals. b) Epifluorescent image (upper panel) of transgenic animals (*dzIs117*, Rahman *et al*. 2022) carrying the indicated transgenes, which label FLP in green and PVD in magenta (image courtesy of M. Trivedi). A schematic tracing is shown (lower panel). c) Schematics of the AVM touch receptor neuron (left) and the PVD mechanosensory neuron (right). Note the stark difference in morphology between the 2 neurons, which is controlled by different levels of the MEC-3/LIM homeobox transcription factor and possibly by different ratios of MEC-3 isoforms as indicated (MEC-3a vs MEC-3d forms). High levels of MEC-3 result in a simple, AVM-like morphology, whereas lower levels result in highly arborized dendritic arbors in PVD (Smith *et al*. 2013). The levels of MEC-3 in PVD are controlled by the MBL-1 splicing factor and the ubiquitin ligase EEL-1 (Xie *et al*. 2023). Several target genes of MEC-3 in PVD are known, including the transcription factor complex EGL-44/EGL-46 (O'Brien *et al*. 2017), the claudin homolog HPO-30 (Smith *et al*. 2013), and the nutrient sensor LET-363/mTOR (Land *et al*. 2023). The EGL-44/EGL-46 and the HPO-30 molecule preferentially coordinate the formation of commissural and pioneer secondary dendrites, respectively.

The dendritic arbors of FLP and PVD neurons cover the surface of the animals in a tiling, nonoverlapping fashion (Fig. 5a and b). Initial ablation experiments indicated that FLP and PVD do not tile through mutual repulsion suggesting that other mechanisms such as molecular or anatomical barriers delineate the establishment of FLP and PVD field size rather than contact-mediated repulsion (Yip and Heiman 2016). However, recent genetic ablation experiments, mutant analyses, and time-lapse experiments suggest that FLP and PVD do tile at least in part through contact-mediated repulsion using mechanisms that involve at least 3 parallel genetic pathways: Netrin-signaling, MIG-14/Wntless, and FMI-1/Flamingo (Trivedi & Bülow, unpublished). These findings are consistent with genetic manipulations that result in the formation of additional PVD neurons, which reveal that duplicated PVD neurons tile through a contact-mediated, Netrin-dependent mechanism (Yip and Heiman 2016). Additional dedicated mechanisms appear to play a role in establishing FLP and PVD field size. For example, a pharynx-derived Wnt signal CWN-2 functions nonautonomously to restrict FLP field size by activating the LIN-17/Frz receptor in FLP neurons (Tzeng and Shen 2023). In conclusion, tiling and the control of field size of PVD and FLP neurons is mediated by both cell-autonomous and cell-nonautonomous mechanisms although more work is required to understand the processes that control patterning of the nonoverlapping sensory fields of FLP and PVD neurons.

## Guided assembly of PVD dendrites

### Transcriptional control of dendrite morphogenesis

**Fig. 6.**
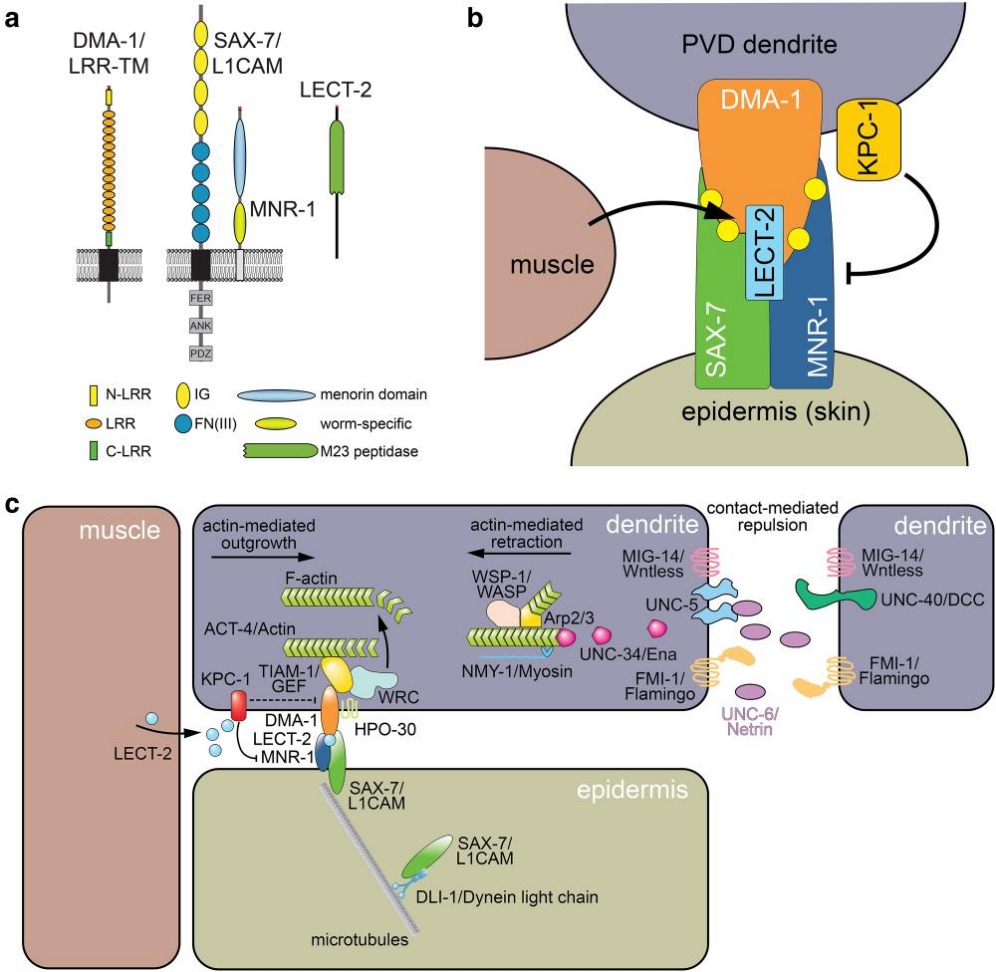
The Menorin complex shapes somatosensory dendrites. a) Schematics of the key factors that form the Menorin complex, including the leucine-rich transmembrane receptor DMA-1, the putative cell adhesion molecules SAX-7/L1CAM and MNR-1/Menorin, and the secreted chemokine LECT-2/Chondromodulin II. b) Schematic of the Menorin complex in the context of different tissues. Yellow circles as part of the Menorin complex indicate *N*-glycosylations of DMA-1 (Rahman *et al*. 2022). c) Shown are various tissues involved and the molecular factors. Central to the interaction between the dendrite and the skin is the Menorin complex, a quaternary complex of the epidermal SAX-7/L1CAM-MNR-1/Menorin complex with the PVD-derived DMA-1/LRR-TM receptor and the muscle-derived chemokine LECT-2/Chondromodulin. The furin-like proprotein convertase KPC-1 inhibits the Menorin complex. It is proposed that signaling downstream of DMA-1/LRR-TM regulates F-actin through at least 2 mechanisms: (1) a direct interaction between DMA-1/LRR-TM, TIAM-1/GEF, and ACT-4/Actin and (2) through an HPO-30-dependent mechanism that involves the wave regulatory complex (WRC). Contact-mediated repulsion between 2 tertiary dendrites is mediated by at least 3 partially redundant pathways: (1) MIG-14/Wntless, (2) Netrin signaling, and (3) the atypical cadherin FMI-1/Flamingo. The retraction of the dendrite is also actin dependent and involves likely retrograde flow in a nonmuscle–myosin-dependent mechanism (see text). ANK, ankyrin-binding domain; FER, FERM-binding domain; PDZ, PDZ domain-binding motif.

Morphogenesis of the FLP and PVD somatosensory dendrites occurs in a stepwise fashion with a combination of overlapping and distinct molecular mechanisms being responsible for different aspects of the developing dendritic arbors. Most work has focused on understanding the patterning of PVD dendrites, but where tested, genes important for PVD patterning serve related functions in FLP or IL2 patterning. Early work on the specification of touch receptor neurons by the Chalfie Lab identified at least 2 homeobox transcription factors that establish PVD and FLP cell fates (Way and Chalfie 1989). The UNC-86/POU homeobox transcription factor functions in a complex with the MEC-3/LIM homeobox transcription factor to directly regulate the expression of *mec-3/LIM* (Xue *et al*. 1992), which in turn through combinatorial use of additional factors controls the specification of PVD neurons. The soft-touch receptor neurons (ALMs, PLMs, AVM, and PVM) are characterized by a simple unbranched dendritic morphology, in contrast to the highly elaborate dendritic trees of PVD somatosensory neurons (Fig. 5c). Each touch neuron class requires a different level of the MEC-3/LIM homeobox transcription factor. Higher levels of MEC-3 are necessary to establish the structure and fate of the simpler soft-touch receptor neurons, while lower levels of MEC-3 result in the more complex dendrites of PVD somatosensory neurons (Smith *et al*. 2013) (Fig. 6c). Somewhat counterintuitively, overexpression of a MEC-3/LIM isoform (MEC-3a) in PVD leads to increased PVD branching (Xie *et al*. 2023). Therefore, additional mechanisms likely play a role to mediate the distinct morphologies of PVD and soft-touch receptor neurons (see below). Regardless, the observations that different levels of the same transcription factor regulate dendrite complexity are conceptually similar to findings in *Drosophila*, where different levels of the transcription factor Cut correspond to different degrees of branching of the da (dendrite arborization) neurons (Grueber *et al*. 2003).

Besides regulation of MEC-3/LIM transcription factor levels in PVD neurons, there likely exists an additional layer of regulation through alternative splicing of the *mec-3/LIM* transcript by the muscle-blind-like (MBNL) splicing factor MBL-1 (Xie *et al*. 2023). MEC-3/LIM normally exists in 2 major splice variants MEC-3a and MEC-3d, and loss of the splicing factor MBL-1/MBNL results in a change of composition with more of the MEC-3d variant at the expense of the MEC-3a variant in whole animals (Xie *et al*. 2023). This results in morphological defects in PVD dendrites, i.e. a simplification of the dendritic arbor while having no effect on the morphology of the AVM and PVM soft-touch receptor neurons (Xie *et al*. 2023). A possible explanation of these findings could be that in addition to the total levels of MEC-3/LIM, the relative composition of splice variants differs between cells. Since MBL-1/MBLN is expressed at 4 times higher levels in PVD compared to AVM/PVM touch receptor neurons (Taylor *et al*. 2021), it is conceivable that MEC-3a is more prevalent in PVD neurons than in AVM/PVM neurons. If MEC-3a is the variant that promotes branching, this would also explain why loss of MBL-1/MBLN, and hence MEC-3a, disrupts morphogenesis of PVD but not AVM or PVM neurons (Fig. 6c). Lastly, a ubiquitin ligase encoded by *eel-1* mediates degradation of MEC-3/LIM providing yet another level of MEC-3/LIM regulation (Xie *et al*. 2023). Together, these posttranscriptional and posttranslational systems coordinate optimal MEC-3/LIM levels (and possibly isoform composition) in PVD neurons to create the elaborate dendritic PVD dendritic arbors (Xie *et al*. 2023) (Fig. 6c). In addition, transcription factors such as the aryl hydrocarbon (dioxin) receptor AHR-1—the *C. elegans* homolog of the basic helix-loop-helix transcription factor Spineless, which controls dendrite branching in *Drosophila—*function in concert with the MEC-3/LIM transcription factor to control PVD dendrite morphogenesis (Smith *et al*. 2013).

Further insight came from cell-specific transcriptomic analyses of PVD neurons, which identified many categories of genes enriched in PVD, including at least 112 transcription factors (Smith *et al*. 2010). Validation of these genes by mutation or RNAi-mediated gene knockdown showed that the transcription factors fall into several classes that control different aspects of PVD morphogenesis, including outgrowth of the primary, secondary, tertiary, and quaternary branches (Smith *et al*. 2010). These experiments also identified transcription factors that normally restrict branch formation (Smith *et al*. 2010). In addition, these studies revealed MEC-3/LIM target genes relevant to PVD, including HPO-30/Claudin (Smith *et al*. 2013) and the TFIIA-like zinc finger transcription factor *egl-46*, which functions with the TEAD transcription factor *egl-44* and regulates development of different types of secondary PVD branches (O'Brien *et al*. 2017). Finally, the nutrient sensor *let-363/ceTOR* was identified as a transcriptional target of MEC-3/LIM, thereby linking developmental programs of dendrite morphogenesis with nutrient availability (Land *et al*. 2023). Other relevant target genes of MEC-3/LIM remain largely unknown. Collectively, these studies strongly suggest that dedicated transcriptional, and by inference, molecular programs exist for individual aspects of PVD patterning.

### Higher-order branches grow along an epidermal molecular scaffold

Progress in understanding of PVD morphogenesis came from a combination of systematic candidate gene as well as unbiased forward genetic approaches. For instance, an unbiased genetic approach to understand PVD morphogenesis used RNAi-mediated knockdown of approximately 3,000 genes on chromosome IV. This study identified 11 genes, including genes encoding for cytoskeletal proteins such as UNC-44/Ankyrin and UNC-70/β-Spectrin, as well as regulators of the actin cytoskeleton such as *gex-2/p140/Sra1*, a member of the wave regulatory complex (WRC) (Aguirre-Chen *et al*. 2011). In addition, these experiments showed that components of MT-based motors such as dynein and kinesin as well as the conserved adapter protein BICD-1/Bicaudal D are important for patterning of PVD dendrites. Specifically, BICD-1/Bicaudal D is important for restricting branching proximal to the PVD cell body (Aguirre-Chen *et al*. 2011). Collectively, these findings demonstrated that mechanisms of dendrite morphogenesis of PVD somatosensory dendrites are conserved with other experimental systems and that the cytoskeleton and molecular motors play a conserved role in dendrite morphogenesis (Aguirre-Chen *et al*. 2011).

Systematic analyses of expression patterns of 17 transmembrane receptors with leucine-rich repeat domains (LRR-TM) encoded in the *C. elegans* genome identified *dma-1* (for *d*endrite *m*orphology *a*bnormal), which displayed predominant expression in PVD and FLP neurons (Fig. 6a and b) (Liu and Shen 2011). Deletion of *dma-1/LRR-TM* resulted in strongly defective PVD and FLP dendrites with essentially no tertiary and quaternary branches and a reduced number of tangled secondary branches (Liu and Shen 2011). DMA-1/LRR-TM functions cell autonomously in PVD and FLP dendrites and is localized both to intracellular vesicles and the plasma membrane (Liu and Shen 2011). Misexpression of DMA-1/LRR-TM in PDE or PLM sensory neurons leads to ectopic branch formation, suggesting that DMA-1/LRR-TM is both necessary and sufficient for dendritic branching (Liu and Shen 2011). Unbiased forward genetic approaches identified additional factors, including the conserved SAX-7/L1CAM and MNR-1/Menorin adhesion proteins (Fig. 6a and b) (Dong *et al*. 2013; Salzberg *et al*. 2013). Mutants in SAX-7/L1CAM and MNR-1/Menorin result in highly disorganized dendritic arbors in PVD neurons due to an inability to form stable tertiary branches (Dong *et al*. 2013; Salzberg *et al*. 2013). Both proteins function from the skin to shape PVD higher-order dendrites. SAX-7/L1CAM is subcellularly localized along a thin line in the skin where the lateral epidermis abuts the lateral muscle quadrants (also referred to as the tertiary line, cf. Fig. 4a–c) (Dong *et al*. 2013; Salzberg *et al*. 2013). While relatively little is known about the mechanisms of subcellular localization of SAX-7/L1CAM, dynein light chain (*dli-1*) functions in the skin to localize SAX-7::GFP to the edge of the lateral epidermis (Zhu *et al*. 2017). This finding implies that minus-end-directed MT-based transport in the skin is part of the underlying molecular mechanism to localize SAX-7/L1CAM to its subcellular localization. In contrast, reporters for the putative MNR-1/Menorin cell adhesion molecule appear expressed in the skin in a punctate pattern (Dong *et al*. 2013; Salzberg *et al*. 2013) and nothing is known about possible mechanisms of its putative surface localization or regulation.

The cell adhesion molecule SAX-7/L1CAM comprises 2 isoforms with either 4 or 6 immunoglobulin (Ig) domains followed by 5 fibronectin III (FN III) repeats in the extracellular domain (Fig. 6a). For PVD patterning, SAX-7/L1CAM requires the FN III domains, and particularly the third FN III domain, but not the Ig domains or the conserved intracellular domain (Dong *et al*. 2013; Salzberg *et al*. 2013). By contrast, in other contexts, such as neuronal maintenance and neurite branching, SAX-7/L1CAM functions require the Ig domains rather than the FN III domains (Pocock *et al*. 2008; Diaz-Balzac *et al*. 2015). For retrograde extension of ciliated dendrites in sensory neurons, the intracellular domain of SAX-7/L1CAM is critically important to mediate interactions with adapters such as MAGI-1/MAGUK that link SAX-7/L1CAM to the actin cytoskeleton (Cebul *et al*. 2024) as discussed in “Ciliated dendrites not ensheathed by glia.” MNR-1/Menorin contains a DUF2181 (domain of unknown function 2181 or Menorin domain), which is conserved from choanoflagellates to humans (Fig. 6a and b). The Menorin domain is important for function because missense mutations in the domain result in developmental defects of PVD dendrites (Salzberg *et al*. 2013). SAX-7/L1CAM and MNR-1/Menorin are part of a biochemical complex, both in vivo and in vitro (Dong *et al*. 2013; Salzberg *et al*. 2013).

In addition to these 2 skin-derived factors, genetic screens also identified LECT-2, a secreted cytokine with homology to leukocyte cell-derived chemotaxin 2 (also named Chondromodulin II) in vertebrates (Fig. 6a) (Diaz-Balzac *et al*. 2016; Zou *et al*. 2016). LECT-2/Chondromodulin II is secreted from muscle and localizes to the lateral lines formed by SAX-7/L1CAM in the skin (cf. Fig. 4c, light blue, dashed line) (Diaz-Balzac *et al*. 2016; Zou *et al*. 2016). Loss of SAX-7/L1CAM results in complete loss of LECT-2/Chondromodulin II localization, suggesting that LECT-2/Chondromodulin II binds to SAX-7/L1CAM on the cell surface (Diaz-Balzac *et al*. 2016; Zou *et al*. 2016). Indeed, SAX-7/L1CAM, LECT-2/Chondromodulin II, MNR-1/Menorin, and DMA-1/LRR-TM can form a high-affinity multiprotein complex that shapes PVD dendrite arborization (Fig. 6b) (Zou *et al*. 2016). LECT-2/Chondromodulin II function as a secreted, diffusible cue because transgenic expression from distant tissues can rescue the mutant defects (Diaz-Balzac *et al*. 2016). However, there is also evidence in vivo that LECT-2/Chondromodulin II may act more locally. When genetic mosaics were used to remove *lect-2* from individual muscle cells, the PVD dendrites overlying those muscle cells appeared less well patterned than dendrites over adjacent muscle cells where LECT-2/Chondromodulin II was retained (Zou *et al*. 2016). Taken together, these studies suggest that LECT-2/Chondromodulin II functions as a permissive cue to allow the formation of the Menorin complex, a high-affinity complex comprising LECT-2/Chondromodulin II, SAX-7/L1CAM, MNR-1/Menorin, and the DMA-1/LRR-TM receptor (Diaz-Balzac *et al*. 2016; Zou *et al*. 2016). More recent genetic and biochemical evidence suggests that the activity of the Menorin complex is further modulated by specific *N-*glycan structures that are attached to the DMA-1/LRR-TM protein on dedicated *N-*glycosylation sites implying additional levels of regulation during dendrite morphogenesis (Fig. 6b) (Rahman *et al*. 2022).

Forward genetic screens also identified *kpc-1/Furin*, which encodes a furin-related proprotein convertase, as an important regulator of FLP and PVD patterning (Schroeder *et al*. 2013; Salzberg *et al*. 2014; Dong *et al*. 2016). Mutations in *kpc-1/Furin* display characteristic defects in PVD arbor formation that are distinct from mutations in the Menorin pathway (Salzberg *et al*. 2014). In addition to patterning defects in higher-order branches of PVD, *kpc-1/furin* mutant animals display self-avoidance defects with tertiary dendrites of PVD failing to retract upon contact (Salzberg *et al*. 2014). Transgenic rescue experiments showed that (1) *kpc-1/Furin* functions cell autonomously in PVD and (2) acts with the Menorin pathway to pattern dendritic arbors. However, these studies also showed that loss of *kpc-1/Furin* can partially suppress PVD patterning defects in *sax-7/L1CAM* or *mnr-1/MNR-1* mutants suggesting that *kpc-1/Furin* serves as a negative regulator of the Menorin pathway (Salzberg *et al*. 2014). Further studies provided a possible molecular explanation for these observations. Loss of *kpc-1/Furin* resulted in an increased localization of DMA-1/LRR-TM to the plasma membrane, and KPC-1/Furin was shown to form a biochemical complex with DMA-1/LRR-TM (Dong *et al*. 2016). These findings were interpreted such that *kpc-1/Furin* is required for endocytosis of the DMA-1/LRR-TM receptor thereby negatively regulating the Menorin pathway (Dong *et al*. 2016). It was further shown that the proteolytic activity of KPC-1/Furin was required for self-processing because a processed form of KPC-1/Furin could rescue the *kpc-1/Furin* mutant phenotype (Dong *et al*. 2016). However, a processed version of KPC-1/Furin in which 2 conserved residues of the catalytic triad were mutated failed to transgenically rescue the defects in *kpc-1* mutants suggesting that KPC-1/Furin processes additional protein targets beyond self-activation (Ramirez-Suarez *et al*. 2023). Interestingly, a modifier screen of a partial loss of function allele of *kpc-1/Furin* identified a partial loss of function allele of *mnr-1/Menorin*, which completely suppressed the hypomorphic *kpc-1* allele (Ramirez-Suarez *et al*. 2023). This mutual suppression was dependent on both residual *kpc-1/Furin* and *mnr-1/Menorin* function suggesting direct functional interactions. Intriguingly, the increased membrane localization of DMA-1/LRR-TM in *kpc-1/Furin* mutants required *mnr-1/Menorin*, suggesting that DMA-1/LRR-TM cell membrane localization is at least in part regulated through MNR-1/Menorin (Fig. 6) (Ramirez-Suarez *et al*. 2023). Consistent with this interpretation, MNR-1/Menorin is also sufficient to regulate DMA-1/LRR-TM surface localization. On the other hand, mutants in *kpc-1/Furin* showed decreased amounts of LECT-2/Chondromodulin II protein (Ramirez-Suarez *et al*. 2023). Taken together, these findings point to more complex mechanisms of how KPC-1/Furin regulates the Menorin pathway that likely go beyond the regulation of DMA-1/LRR-TM receptor endocytosis and may include regulatory mechanisms *in trans* (Fig. 6).

### The primary branch grows along an axon scaffold

**Fig. 7.**
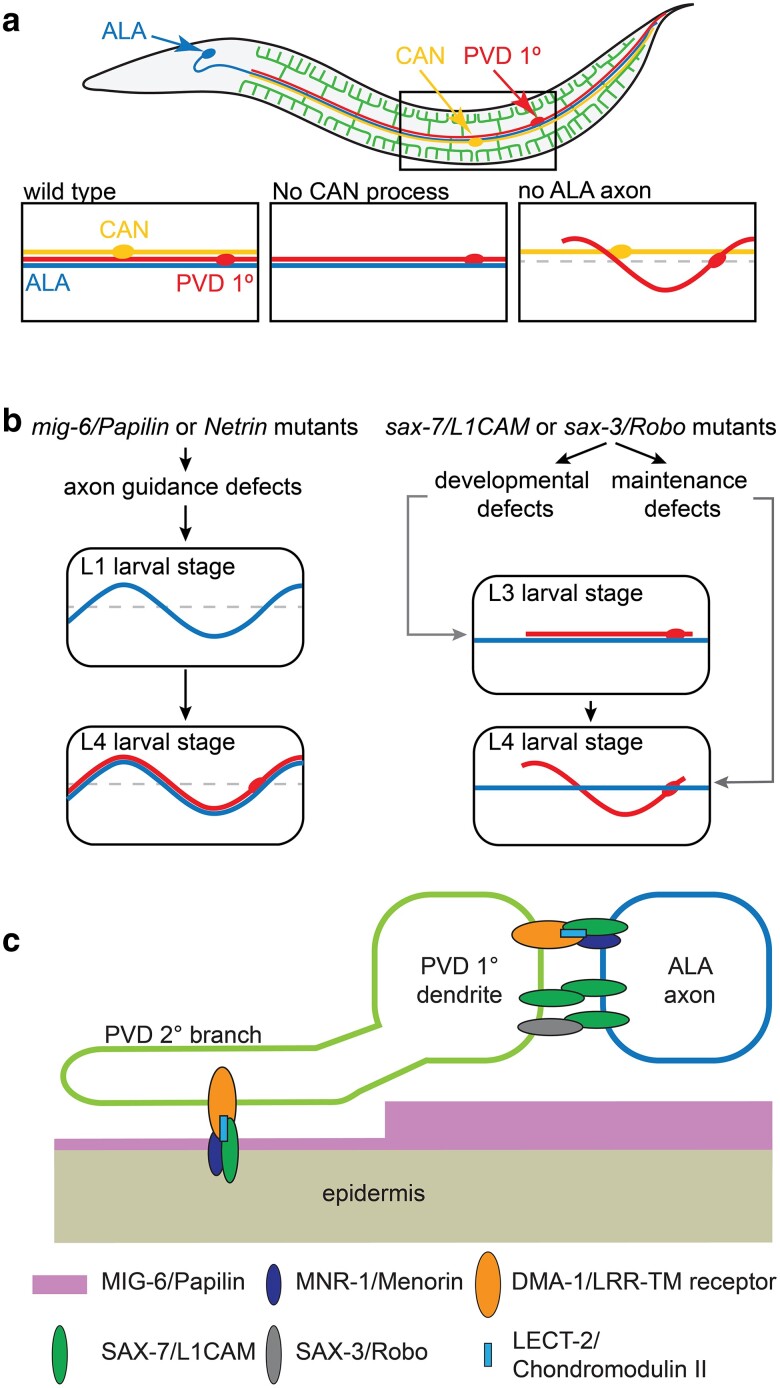
Coordinated assembly of primary PVD dendrites. a) Schematic of the lateral nerve tract comprising a process from ALA, CAN, and the primary PVD dendrite. Ablation of ALA, but not CAN, results in PVD primary dendrite defects. b) Several genes mediate guidance of the ALA process, which serves as the scaffold for the PVD primary dendrite, including the extracellular matrix protein MIG-6/Papilin and the Netrin axon guidance pathway. c) The ALA axon serves as a scaffold for the outgrowing PVD dendrites, which is mediated by the Menorin pathway. SAX-7/L1CAM homodimers and SAX-7/L1CAM-SAX-3/Robo heterodimers maintain fasciculation of the axodendritic bundle (Chen *et al*. 2019; Ramirez-Suarez *et al*. 2019). Secondary dendrites grow out by using the Menorin pathway. Schematics modified from Ramirez-Suarez *et al*. (2019).

The primary dendrite branch of PVD grows out during larval stages along a bundle of processes comprised of the ALA neuron, the CAN cells, and the excretory canal, all structures that form during embryogenesis (White *et al*. 1986). Ablation experiments showed that the processes of ALA but not CAN are required for both guidance and extension of PVD primary dendrites (Chen *et al*. 2019; Ramirez-Suarez *et al*. 2019) (Fig. 7a). ALA guidance is mediated by the Netrin pathway and requires the conserved extracellular matrix molecule MIG-6/Papilin. Therefore, mutations in these genes result in defects in ALA and, consequently, in PVD primary dendrite patterning (Ramirez-Suarez *et al*. 2019) (Fig. 7b). Extension of the PVD primary dendrite is mediated by the Menorin pathway, with *mnr-1/Menorin* and *sax-7/L1CAM* expressed in ALA neurons as the substrates for the PVD-expressed DMA-1/LRR-TM receptor (Ramirez-Suarez *et al*. 2019) (Fig. 7c). At the same time, fasciculation between ALA axons and PVD dendrites appears to be mediated by both homophilic interactions of SAX-7/L1CAM and heterophilic interactions between SAX-7/L1CAM and SAX-3/Robo, both of which are also expressed in PVD dendrites (Fig. 7c) (Chen *et al*. 2019; Ramirez-Suarez *et al*. 2019). In conclusion, the primary dendrite is patterned using the ALA axons rather than the skin as a scaffold, employing both similar and also distinct mechanisms compared to the mechanisms that shape higher-order branches.

### ECM and other factors

Several other extracellular factors have been implicated in dendrite morphogenesis. For example, the conserved heparan sulfate proteoglycan UNC-52/Perlecan has been suggested to be involved in localizing the stripes of SAX-7/L1CAM that organize the formation of quaternary PVD dendrites (Liang *et al*. 2015). Normally, UNC-52/Perlecan mediates the transcellular interaction between the dense bodies in muscle and the intermediate filaments in the skin, which is crucial for correct locomotion (Rogalski *et al*. 1993; Rogalski *et al*. 1995). Liang *et al*. reported that a SAX-7::GFP reporter is localized to stripes that are interspersed with the stripes of UNC-52/Perlecan and that defects in muscle structure due to mutations in *unc-52/Perlecan* result in defects in quaternary branch patterning of PVD dendrites. On the other hand, UNC-52/Perlecan has been described to serve a role in controlling the number of secondary branches. This function relies on 4 conserved Ig domains present in perlecan genes across phyla and is genetically separable from muscle defects observed in *unc-52/Perlecan* mutants (Celestrin *et al*. 2018). The 4 Ig domains are necessary for correctly localizing the extracellular matrix protein NID-1/Nidogen. Genetically, both *nid-1/Nidogen* and *unc-6/Netrin*, which encodes the secreted netrin cue UNC-6/Netrin, function with *unc-52/Perlecan* to regulate the number of secondary branches (Celestrin *et al*. 2018). One possible explanation is that UNC-52/Perlecan in conjunction with NID-1/Nidogen controls localization, presentation, or diffusibility of the UNC-6/Netrin ligand, which is recognized by the UNC-40/DCC netrin receptor in PVD dendrites (Celestrin *et al*. 2018). The different findings for *unc-52/Perlecan* mutants with regard to secondary and quaternary branches will require additional experimentation to resolve.

### Self-avoidance and mechanisms that restrict dendrite morphogenesis

As also discussed above in “Maintenance, aging, and degeneration of ciliated dendrites,” the mechanisms that promote dendrite growth are complemented by a dedicated set of mechanisms that serve to restrict excessive dendrite growth and branching. Dendrites in general, and PVD dendrites in particular, face the problem that growing dendrites should avoid overlapping in order to cover a territory efficiently, a phenomenon referred to as self-avoidance. In the highly ordered PVD neurons, this developmental challenge becomes most apparent in the tertiary dendrites that grow out in a posterior and anterior direction but should not touch the tertiary dendrites of an adjacent menorah. Time-lapse studies showed that following a brief touch, both tertiary dendrites retract implying a contact-mediated repulsive mechanism (Smith *et al*. 2010). Several genes have been identified that are required for self-avoidance of PVD tertiary dendrites, including the secreted UNC-6/Netrin cue. A model was proposed where the netrin receptor UNC-40/DCC on the tip of a tertiary dendrite captures UNC-6/Netrin, leading to a repulsive interaction through the UNC-5 netrin receptor on an opposing tertiary dendrite (Fig. 6c) (Smith *et al*. 2012). Studies using mutants that have multiple ectopic PVD neurons spaced evenly along the body axis found that the ectopic dendrite arbors occupy distinct territories (“tiling”) through mutual avoidance in a manner dependent on UNC-6/UNC-40/UNC-5 (Yip and Heiman 2016). Some of the genes that function downstream of UNC-6/Netrin are involved in regulating actin polymerization, such as *unc-34/ENA-VASP*, mutants in which show comparable defects in self-avoidance of tertiary branches (Fig. 6c) (Sundararajan *et al*. 2019a). Similarly, other actin regulators such as the guanine nucleotide exchange factor (GEF) *unc-73/TRIO* and *mig-10/Lamellipodin* regulate self-avoidance without affecting general PVD outgrowth (Liao *et al*. 2018; Sundararajan, *et al*. 2019a). A possible explanation for the observation that mutations in factors that promote actin polymerization result in defects in dendrite self-avoidance, i.e. a failure to retract the dendrite, is the finding that mutations in nonmuscle myosin show similar defects in self-avoidance of tertiary branches (Fig. 6c) (Sundararajan *et al*. 2019a). Therefore, nonmuscle myosin may promote retraction through the promotion of retrograde flow.

Mutations in several additional genes result in self-avoidance defects in tertiary PVD dendrites, including MIG-14/Wntless, a conserved molecule necessary for the secretion of Wnt ligands. Curiously, the function in self-avoidance is independent of Wnts suggesting that MIG-14/Wntless may function by a novel mechanism (Fig. 6c) (Liao *et al*. 2018). Similarly, mutations in the atypical cadherin FMI-1/Flamingo result in self-avoidance defects, likely through misregulation of the actin cytoskeleton (Hsu *et al*. 2020). The FMI-1 pathway functions genetically in parallel to both the Netrin and MIG-14/Wntless pathways, suggesting that self-avoidance is a mechanism that is regulated by highly redundant genetic mechanisms (Fig. 6c) (Liao *et al*. 2018; Hsu *et al*. 2020).

Generally, mechanisms that restrict branching are much less understood with few exceptions. Mutations in the fusogen *eff-1* display phenotypes with excessive branching of dendrites (Oren-Suissa *et al*. 2010; Zhu *et al*. 2017), although there are conflicting results regarding where *eff-1* functions to restrict branching. In one possible model, *eff-1* acts cell autonomously to prune excessive dendrites through autofusion and fission (Oren-Suissa *et al*. 2010). In an alternative model, *eff-1* functions nonautonomously in the skin. In *eff-1* mutants, the skin shows fusion defects with abnormal cell junctions, along which the SAX-7/L1CAM ligand is mislocalized, leading to the aberrant growth of PVD dendrites (Zhu *et al*. 2017). Additional experiments will be required to reconcile these seemingly contradictory findings. Recently, *rabr-1*, which encodes an atypical Rab-related protein, was identified and shown to act in the skin to restrict PVD dendrite branching (Salazar & Bülow, in press). Intriguingly, *rabr-1* mutants do not display any defects in epidermal morphology leaving a possible relationship with *eff-1* unclear. On the other hand, recent studies found a role for the kinetochore protein KNL-1 and its associated KMN (Knl1/Mis12/Ndc80 complex) network, in addition to their role in ciliated dendrite morphogenesis (see section “Glial-ensheathed dendrites are arranged in sense organs”), also function autonomously in PVD neurons to specifically restrict the formation of quaternary branches (Green *et al*. 2023). Unexpectedly, this is mediated through the regulation of the actin cytoskeleton at the tips of developing dendrites (Green *et al*. 2023). Collectively, these findings indicate that dedicated pathways exist in different tissues to restrict dendrite branching at various stages during morphogenesis.

## Cell-intrinsic control of PVD dendrite morphogenesis

### Genes involved in proteostasis and trafficking

A role for proteostasis in PVD morphogenesis was revealed by the identification of mutations in *ire-1* as an important factor for PVD and FLP morphogenesis (Wei *et al*. 2015; Salzberg *et al*. 2017). Inositol-requiring enzyme type 1 (IRE-1) is a major sensor that regulates the unfolded protein response across phyla and comprises both nuclease and kinase activity (Walter and Ron 2011). Activation of IRE-1 results in autophosphorylation, which activates the nuclease activity to create a functional transcript of the *xbp-1* transcription factor by unconventional splicing. The XBP-1 transcription factor initiates a transcriptional program that alleviates protein stress in the endoplasmic reticulum (ER). PVD development requires both the nuclease and kinase activity of IRE-1, although splicing of *xbp-1* appears dispensable (Wei *et al*. 2015; Salzberg *et al*. 2017). Interestingly, dendrite morphogenesis of dissociated rat hippocampal neurons also required IRE1 function, suggesting that the function of IRE1 during dendrite patterning is conserved from worms to mammals (Salzberg *et al*. 2017). Rather than acting through the canonical *xbp-1* pathway, *ire-1* likely functions through the alternative regulated Ire1-dependent decay (RIDD) pathway, a notion that was supported by the finding that cell-specific CRISPR-mediated inactivation of the *xrn-1* RNA endonuclease that is involved in the process resulted in similar phenotypes in PVD morphogenesis as in *ire-1* mutants (Wei *et al*. 2015). In *ire-1* mutants, the DMA-1/LRR receptor becomes trapped in the ER, a phenotype that can be rescued by overexpression of the *hsp-4* chaperone (Wei *et al*. 2015). The defects in PVD dendrite morphogenesis in *ire-1* mutants can also be rectified by reducing insulin signaling (Salzberg *et al*. 2017). A possible explanation for this observation is that reduced insulin signaling has been shown to improve ER homeostasis in *ire-1* mutants (Safra *et al*. 2014), thereby possibly removing the secretory block of DMA-1/LRR-TM.

### The role of the cytoskeleton

Neurons are highly polarized cells, and MTs play crucial roles in establishing and maintaining polarity. MTs are polymers of alpha- and beta-tubulin dimers, which polymerize from the so-called minus end to the plus end. A fluorescent reporter for alpha-tubulin transgenically expressed in PVD neurons revealed strong MT staining in PVD axons and primary dendrites, with less staining in higher-order branches (Maniar *et al*. 2012; Tang *et al*. 2019; Sundararajan, *et al*. 2019a). Based on a plus-end MT marker (EB2::GFP), the PVD axon displays a plus-end-out polarity; i.e. polymerization proceeds from the cell body toward the axonal periphery (Taylor *et al*. 2015). In contrast, the anterior PVD dendrite displays a minus-end-out polarity as is common in other species as well, whereas the posterior PVD dendrite displays a plus-end-out orientation like in axons (Taylor *et al*. 2015). Therefore, anterior and posterior primary PVD dendrites are molecularly distinct. The minus-end-out orientation in dendrites is not unique to the anterior PVD dendrite and also found in dendrites of other neurons, e.g. motor neurons (Yan *et al*. 2013).

How is the minus-end-out polarity established in PVD anterior dendrites? Recent work showed that a γ-tubulin ring complex (γ-TuRC)-based MTOC is localized to the growth cone of the outgrowing anterior dendrite (Liang *et al*. 2020). This is reminiscent of the centriolar MTOCs positioned at the distal ends of developing ciliated dendrites and the growing dendrite tip of PQR (see sections “Development of glial-ensheathed sensory dendrites in the amphid” and “Development of glial-ensheathed dendrites in other sense organs”). However, in contrast to these examples, the MTOC in PVD is noncentriolar. Further, unlike the PQR MTOC that lags the growing tip, in PVD, the MTOC is required for dendritic outgrowth and provides a platform for retrograde polymerization of MTs and the establishment of the minus-end-out configuration (Liang *et al*. 2020). At the same time, short plus-end-out MTs are formed from the MTOC in an anterograde direction, i.e. the direction of the distal dendrite growth cone. These short plus-end-out MTs are believed to serve as tracks for *unc-116/kinesin-1-*dependent further distal transport of the MTOC. The MTOC colocalizes with RAB-11-positive endosomal vesicles, which cluster the γ-TuRC as the nucleation center for MT growth near the growth cone in a dynein-dependent process (Liang *et al*. 2020). Two conserved MT minus-end-associated proteins, PTRN-1/Patronin, and NOCA-2/Ninein function redundantly to localize MTOC-related, RAB-11.1-positive vesicles to the growth cone of the anterior PVD dendrite (He *et al*. 2022). In addition, in more mature dendrites, MT polarity may be stabilized via other, MTOC-independent, mechanisms. For example, a recent study showed that anchoring MTs to the cell cortex through a protein complex comprising UNC-44/Ankyrin, UNC-119, and UNC-33/CRMP is important to maintain MT polarity in the PVD primary dendrite (He *et al*. 2020).

The polarity of MTs is clearly important for PVD dendrite morphogenesis, because mutations in MT-dependent motors result in defects in dendrite formation. For example, mutations in the adaptor protein *bicd-1/BicD* or minus-end-directed motor proteins (such as *dhc-1/Dynein heavy chain*) show excessive branching proximal to the cell body with reduced branching in the distal portion of PVD dendrites, possibly because branching relevant cargo such as DMA-1/LRR-TM and HPO-30/Claudin cannot be transported along MTs to the distal parts of the dendrites (Aguirre-Chen *et al*. 2011; Taylor *et al*. 2015). Somewhat nonintuitively, mutations in *unc-116/*kinesin, a plus-end-directed motor, result in similar PVD branching defects (Aguirre-Chen *et al*. 2011; Taylor *et al*. 2015). A possible explanation for these observations is that in *unc-116/kinesin1* mutants, the anterior PVD dendrite shows a reversed plus-end-out MT orientation. Conversely, mutations in the small GTPase *rab-10* result in the opposite phenotype, i.e. excessive distal branching with reduced branching proximal to the cell body of PVD (Taylor *et al*. 2015; Zou *et al*. 2015). Perhaps, *rab-10* functions in trafficking such that, in its absence, branching factors like DMA-1/LRR-TM and HPO-30/Claudin are not localized to the membrane anymore and instead accumulate in intracellular vesicles (Zou *et al*. 2015).

Early screens for genes involved in PVD patterning identified *gex-2/p140/Sra1*, a component of the WRC and *unc-115/LIM*, an actin-binding LIM-domain containing protein, pointing to the importance of the actin cytoskeleton (Aguirre-Chen *et al*. 2011). When visualized in vivo, F-actin was shown to localize to PVD dendrites, specifically to the distal tips of developing dendrites (Zou *et al*. 2018; Tang *et al*. 2019). The terminal quaternary dendrites of PVD are a mere 30–60 nm in diameter, barely wide enough to accommodate a single ∼25-nm-wide MT. Therefore, perhaps not surprisingly, actin filaments (which are each only ∼7-nm wide) play a more important role in patterning the higher-order branches of PVD. Localization of F-actin to growing dendrites was dependent on the activity of the Menorin pathway, i.e. DMA-1/LRR-TM, SAX-7/L1CAM, and MNR-1/Menorin (Tang *et al*. 2019).

Insight into how signaling downstream of the DMA-1/LRR-TM receptor in PVD dendrites functions came again from forward genetic approaches. Analyses of mutations in the conserved GEF *tiam-1/GEF* and *act-4/actin* indicated that DMA-1/LRR-TM functions genetically in a pathway with both factors. TIAM-1/GEF could form a complex with DMA-1/LRR-TM via a PDZ-binding domain in TIAM-1/GEF and a PDZ motif at the C-terminus of DMA-1/LRR-TM (Fig. 6c) (Zou *et al*. 2018; Tang *et al*. 2019). Biochemical and imaging experiments suggested that DMA-1/LRR-TM functions in a complex with the HPO-30/Claudin-like molecule at the cell membrane of PVD dendrites. DMA-1/LRR-TM was proposed to regulate actin polymerization by recruiting the WRC through the C-terminus of HPO-30/Claudin (Zou *et al*. 2018). This could initiate polymerization of branched actin, after which the WRC recruits regulators of linear actin polymerization such as UNC-34/ENA/VASP and UNC-115/abLIM that lead to dendrite extension (Fig. 6c) (Shi *et al*. 2021). HPO-30/Claudin may also bind and regulate actin directly independently of the WRC (Kramer *et al*. 2023). On the other hand, TIAM-1/GEF also interacted directly with ACT-4/Actin in a complex and appeared to function independently of its GEF activity (Fig. 6c) (Tang *et al*. 2019), which had previously been shown to be important for neuronal branching in *C. elegans* and vertebrates (Tolias *et al*. 2005; Demarco *et al*. 2012). Therefore, different pathways downstream of DMA-1/LRR-TM seem to regulate different aspects of PVD patterning, one through HPO-30/Claudin and the WRC and one through TIAM-1/GEF and ACT-4/Actin. Whereas the DMA-1/TIAM-1/HPO-30/WRC branch of the pathway appears to function primarily during the formation of secondary and tertiary PVD dendrites, the DMA-1/TIAM-1/ACT-4 branch of the pathway may be particularly important for the formation of higher-order quaternary branches (Zou *et al*. 2018; Tang *et al*. 2019). This model is supported by 2 observations. A *dma-1* allele missing only the last 4 amino acids, i.e. the PDZ motif that mediates the interaction between DMA-1/LRR-TM and TIAM-1/GEF, results in PVD dendrites with largely normal secondary and tertiary branches but a greatly reduced number of quaternary branches. Conversely, *tiam-1* constructs lacking the PDZ domain but retaining the GEF domain rescued the formation of secondary and tertiary branches but not quaternary branches in *tiam-1/GEF* null mutants (Tang *et al*. 2019). Recently, cortical actin has been shown to be important for dendrite patterning during larval stages and to maintain dendrite integrity during adult stages (Zhao *et al*. 2022). Collectively, these studies reveal a range of complex functions for the actin cytoskeleton during various aspects of dendrite development and maintenance.

### The role of endoplasmic reticulum proteins

Several recent studies have underscored the importance of proteins of the endoplasmic reticulum for PVD development. For example, mutations in the *catp-8/P5A-type ATPase* were identified in screens for defects in PVD morphology (Feng *et al*. 2020; Qin *et al*. 2020; Tang *et al*. 2021), The *catp-8/P5A-type ATPase* encodes an ER-resident enzyme with poorly defined functions that is conserved from plants to vertebrates. These studies revealed the first functions for this highly conserved gene in metazoans and established that CATP-8 can function to localize transmembrane proteins in the secretory pathway in PVD dendrites, including DMA-1/LRR-TM and HPO-30/Claudin, likely by regulating membrane insertion (Feng *et al*. 2020; Qin *et al*. 2020; Tang *et al*. 2021). CATP-8 was also shown to remove mistargeted mitochondrial proteins (Qin *et al*. 2020). Interestingly, *catp-8* serves additional functions in the secretion of different factors in tissues other than PVD to regulate neuronal patterning, including *egl-20/Wnt*, which is important for neuronal migrations (Li *et al*. 2021; Tang *et al*. 2021).

In a screen for genes regulating the morphology of the ER in PVD dendrites, mutations in *atln-1/Atlastin* were isolated (Liu *et al*. 2019). *atln-1/Atlastin* is necessary for the formation and localization of ER networks in PVD dendrites, particularly at dendrite branch sites. The defective ER networks resulted in defects in mitochondrial fission as well as MT stability, which could be secondary to the ER defects (Liu *et al*. 2019). In addition, the CLI-1/inositol 5-phosphatase INPP5K serves conserved functions in fine-tuning tubular ER structure, including in PVD dendrites (Dong *et al*. 2018). While both mutations in *atln-1/Atlastin* or *cli-1*/*INPP5K* affected ER morphology in PVD neurons, neither gene seemed to play a visible role in PVD morphogenesis although more subtle or redundant functions could not be excluded.

## Plasticity of dendritic arbors

### Plasticity during aging

The dendrite morphology of PVD dendrites is not uniform and static. For instance, animals raised in isolation and therefore without mechanical stimulation from contact with other individuals show increased levels of dendrite branching (Inberg and Podbilewicz 2018; Inberg *et al*. 2022). These animals also display differences in their sensitivity to harsh touch following rearing with or without mechanical stimulation. Both the morphological and the behavioral changes depend on a distinct combination of mechanosensory amiloride-sensitive epithelial sodium channels (Inberg and Podbilewicz 2018; Inberg *et al*. 2022). Therefore, the dendritic arbors of PVD display considerable plasticity and can be influenced by environmental experience. Additionally, PVD dendrites display increasing arbor complexity as well as increasing self-avoidance defects as animals age (Kravtsov *et al*. 2017) further showing that PVD dendritic arbors are not fixed but can be remodeled by either experience during development or aging.

### Degeneration

In addition to the age-related increases in dendrite branching, PVD dendrites in aging animals display a range of other abnormalities, including the appearance of bead/bubble-like structures and the fragmentation of MTs through an autophagy-dependent mechanism (E *et al*. 2018). The age-related defects are initiated by the G-protein-coupled receptor NPR-12 in PVD, which is activated non-autonomously by the antimicrobial peptide NLP-29, which is expressed from the skin (E *et al*. 2018). In addition, proteins involved in MT-based mitochondrial transport have been shown to be important to prevent age-related degeneration of PVD dendrites (Zhao *et al*. 2021). Moreover, mutations in the calponin homology domain-containing protein CHDP-1 also result in dendrite degeneration due to effects on cortical actin and MT stability. Taken together, these findings underscore the importance of the cytoskeleton for the maintenance of dendrite morphology during aging.

### Regeneration

Lastly, PVD dendritic arbors have been used to study dendrite regeneration following dendrotomy. When the dendritic branches of PVD are severed by a laser, the majority of dendrites reconnect. Surprisingly, this process is not dependent on the fusogen *eff-1* (Oren-Suissa *et al*. 2017), which cell autonomously mediates axonal fusion after axotomy (Ghosh-Roy *et al*. 2010). Instead, a second fusogen, encoded by the *aff-1* gene, mediates PVD regeneration following dendrotomy but unexpectedly appears to act in the skin to nonautonomously promote dendrite regeneration (Oren-Suissa *et al*. 2017). Overexpression of AFF-1 is also able to reinstate regenerative potential in aging PVD dendrites. A recent more expanded study of genes involved in axon regeneration also demonstrated that the genes that mediate axon regeneration are not involved in dendrite regeneration (Brar *et al*. 2022). This study also showed that the Rac GTPase CED-10 and the guanine exchange factor TIAM-1/GEF function upstream and cell autonomously in PVD dendrites to mediate regeneration. Therefore, distinct molecular mechanisms control axonal and dendritic regeneration. Future work is expected to shed light on these important but less studied aspects of dendrites.

## Concluding remarks

The molecular and genetic mechanisms that shape the diversity of dendritic morphologies are molecularly complex and require distinct but overlapping sets of genes. Common to the processes described here is the importance of different surrounding tissues, including skin, muscle, glia, and other neurons, all of which contribute specific factors that function in concert during dendrite morphogenesis. “It takes a village to raise a dendrite” could describe this phenomenon. Not surprisingly, *C. elegans* with its powerful genetic tools has been instrumental to unravel the conserved genes and mechanisms that mediate these interactions between the tissues. A focus for the future will be to better understand the cell biological processes that execute morphogenesis and that regulate the subcellular localization of different factors in different tissues. The worm, no doubt, will likely be at the forefront of these discoveries once again.
